# ICU patients receiving remifentanil do not experience reduced duration of mechanical ventilation: a systematic review of randomized controlled trials and network meta-analyses based on Bayesian theories

**DOI:** 10.3389/fmed.2024.1370481

**Published:** 2024-08-07

**Authors:** Fangjie Lu, Sirun Qin, Chang Liu, Xunxun Chen, Zhaoqiu Dai, Cong Li

**Affiliations:** ^1^Department of Critical Care Medicine, Southern University of Science and Technology Yantian Hospital, Shenzhen, Guangzhou Province, China; ^2^Department of Critical Care Medicine, Changshu Hospital Affiliated to Nanjing University of Traditional Chinese Medicine, Changshu, Jiangsu, China; ^3^Department of Cardiovascular Medicine, The Third Xiangya Hospital of Central South University, Changsha, Hunan, China; ^4^Department of Emergency Center, Affiliated Huaian Hospital of Xuzhou Medical University, Huaian, China; ^5^Center for Tuberculosis Control of Guangdong Province, Guangzhou, China; ^6^Department of Traditional Chinese Medicine, Changshu Hospital Affiliated to Nanjing University of Traditional Chinese Medicine, Changshu, Jiangsu, China

**Keywords:** critical illness, mechanical ventilation, analgesics, opioid, remifentanil, network meta-analysis

## Abstract

**Background:**

The purpose of this network meta-analysis (NMA) was to evaluate the efficacy of intravenous opioid μ-receptor analgesics in shortening the duration of mechanical ventilation (MV) in ICU patients.

**Methods:**

Randomized controlled trials comparing the efficacy of remifentanil, sufentanil, morphine, and fentanyl on the duration of MV in ICU patients were searched in Embase, Cochrane, Pubmed, and Web of Science electronic databases. The primary outcome was MV duration. The Bayesian random-effects framework was used to evaluate relative efficacy.

**Results:**

In total 20 studies were included in this NMA involving 3,442 patients. Remifentanil was not associated with a reduction in the duration of MV compared with fentanyl (mean difference (MD) -0.16; 95% credible interval (CrI): −4.75 ~ 5.63) and morphine (MD 3.84; 95% CrI: −0.29 ~ 10.68). The secondary outcomes showed that, compared with remifentanil, sufentanil can prolong the duration of extubation. No regimen significantly shortened the ICU length of stay and improved the ICU mortality, efficacy, safety, and drug-related adverse events.

**Conclusion:**

Among these analgesics, remifentanil did not appear to be associated with a reduction in MV duration. Clinicians should carefully titrate the analgesia of MV patients to prevent a potentially prolonged duration of MV due to excessive or inadequate analgesic therapy.

**Systematic Review Registration:**

https://www.crd.york.ac.uk/prospero/, CRD42021232604.

## Highlights

- Question: Is remifentanil more effective than other intravenous opioid analgesics at reducing mechanical ventilation duration in ICU patients?
- Findings: Compared to other intravenous analgesics that target the μ-receptor opioid, Remifentanil did not show any decrease in the length of mechanical ventilation.
- Meaning: To prevent excessive or inadequate analgesia prolonging the duration of mechanical ventilation, clinicians are advised to carefully titrate analgesia.

## Introduction

### Description of the intervention

Intensive care unit (ICU) patients on invasive mechanical ventilation (MV) experience pain, especially in patients requiring long-term MV (1–4). These unpleasant sensory experiences may prevent MV weaning (5). Therefore, preemptive analgesic therapy should be administered to ICU patients on MV to alleviate pain (5–7).

Intravenous (IV) opioid μ-receptor analgesics, such as morphine, fentanyl, and sufentanil, are considered first-line drugs for the treatment of nonneuropathic pain (5, 7, 8). However, they have long half-lives and are easily redistributed and accumulated. Even when administered at doses normally used for several days, they are associated with increased respiratory depression and prolonged duration of MV (5, 9–17). Hence, the use of fentanyl, sufentanil, and morphine should be restricted to mechanically ventilated patients requiring long-term analgesia (4, 7, 18).

Remifentanil is a potent selective μ-opioid receptor that is rapidly metabolized by non-specific esterases into inactive metabolites (19, 20). As a result, regardless of the dose and duration of infusion, its onset and offset are very rapid and its context-sensitive half-life is extremely short (19–21). Therefore, remifentanil can be easily titrated and administered for prolonged periods, with a lower risk of respiratory depression (5, 10, 22). It seems to make remifentanil more ideal for ventilated ICU patients.

### Controversy of the intervention

The advantages of remifentanil in reducing the duration of MV in ICU patients have been debated. Randomized controlled trials (RCTs) have examined that the MV duration for remifentanil-based analgesia was significantly shorter than that for morphine-based, fentanyl-based, and sufentanil-based analgesia in postsurgical patients and patients undergoing MV for up to 10 days (23–26). Similarly, remifentanil reduced MV duration in these patients when compared with other opioid μ-receptor analgesics, according to two meta-analyses (27, 28). Even so, opioids administered intravenously at similar pain intensity endpoints seem to exhibit similar MV durations (5). Analgesia with remifentanil had a similar duration of MV as that with fentanyl or morphine when used in postsurgical and non-surgical mechanically ventilated patients and NICU patients undergoing MV for up to 5 days (29–31). In addition, a meta-analysis including 1,067 critically ill patients showed that remifentanil was not associated with a significantly shorter duration of MV than other opioids (32). Moreover, the majority of RCTs have even shown an increased risk of hypotension and bradycardia (25, 33).

### Importance of study

No network meta-analysis (NMA) has evaluated the efficacy of intravenous opioid μ-receptor analgesics in shortening the duration of MV in ICU patients. In view of the uncertainty surrounding sufentanil, fentanyl, morphine, and remifentanil’s efficacy in shortening the duration of MV, we designed this systematic review and NMA to evaluate and rank their effectiveness in reducing MV duration among ICU patients. In addition, the efficacy of these drugs on clinically important outcomes and drug-related adverse events (AEs) was also investigated.

## Methods

### Approval

This article complies with the PRISMA statement (34). The registration number of PROSPERO was CRD 42021232604.

### Eligibility criteria

#### Types of studies, participants, and interventions

In this NMA, we included only full-text published RCTs that involved 16-year-old ICU patients undergoing invasive MV via endotracheal intubation. Studies comparing two or more of the four therapies were included (remifentanil, sufentanil, fentanyl, and morphine).

#### Types of outcome measures

As a primary outcome, the duration of MV was evaluated. Secondary outcomes included extubation duration, ICU mortality, ICU length of stay (LOS), safety, drug-related bradycardia, drug-related hypotension, and drug-related bradycardia.

#### Exclusion criteria

Studies with controlled before-and-after comparisons, interrupted time series studies, and controlled clinical trials were excluded from our analysis. A study without reporting outcome variables, or a study with duplicate publications, was excluded from the study.

### Search strategy

#### Electronic searches

Electronic medical databases including PubMed, Embase, Web of Science, and Cochrane were systematically searched for clinical trials published from 1 January 1991 to 31 December 2023. No language restrictions were applied. Each database used specific search terms, and the search strategy details (Supplementary File 1) were developed as proposed by Cochrane (35). We searched relevant literature using the following MeSH terms and their entry terms: ‘Critical Care’ OR ‘Critical Illness’ OR ‘Intensive Care Units’ OR ‘Coronary Care Units’ OR ‘Respiratory Care Units’ OR ‘Postoperative Care’ OR ‘Burn Units’ AND ‘Respiration, Artificial’ OR ‘Ventilators, Mechanical’ OR ‘Liquid Ventilation’ OR ‘active Ventilatory Support’ OR ‘Continuous Positive Airway Pressure’ OR ‘Intermittent Positive-Pressure Breathing’ OR ‘Positive-Pressure Respiration’ OR ‘High-Frequency Ventilation’ OR ‘Airway Extubation’ OR ‘Intubation, Intratracheal’ AND ‘Analgesics, Opioid’ OR ‘Analgesics’ OR ‘Remifentanil’ OR ‘Sufentanil’ OR ‘Fentanyl’ OR ‘Morphine’.

#### Searching other resources

Our search for relevant gray literature was conducted via Google Scholar. We also searched the following registers for complete trials (latest search 31 December 2023): ISRCTN,^1^ World Health Organization International Clinical Trials Registry Platform (ICTRP),^2^ Chinese Clinical Trial Register,^3^ and ClinicalTrials.gov.

### Data collection and analysis

#### Study selection

Abstracts and titles of selected articles were independently reviewed by four reviewers. Thereafter, they carefully read the full text and decided to include studies. When there were any discrepancies between the reviewers, it was necessary to discuss them with the fifth reviewer and make a decision after consensus.

#### Definition of interventions and outcomes

All study drugs included in this study were IV opioid μ-receptor analgesics. The duration of MV was defined as the time from administration of the study drug after the patients were randomized into groups until the time of actual extubation. The extubation duration was defined as the time from the patient meeting the extubation criteria to the actual extubation. Safety was defined as the occurrence of drug-related AE. Drug-related AE included drug-related hypotension, drug-related bradycardia, and drug-related bradypnea. If AE was not specified as drug-related, it was presumed to be related. In the definition of drug-related hypotension, mean arterial pressure was multiplied by 50 millimeters of mercury. In the definition of drug-related bradycardia, the heart rate was multiplied by 50 beats per minute. In the definition of drug-related bradypnea, the respiratory rate was multiplied by 12 breaths per minute. The criteria for the MV model, weaning from MV, and extubation are shown in Supplementary File 2.

#### Data extraction

The Cochrane Handbook was used to collect all the data. Using the data from the study, five investigators extracted details of the study (language, published year, author, institutions, and funding), participant information (gender and age range), intervention information (drug, duration, and route of administration), results (MV duration and secondary outcomes), and methodological design (randomization, blinding, and allocation concealment) from each study. When there were any discrepancies between the reviewers, it was necessary to discuss and make a decision after consensus with the sixth reviewer.

#### Risk of bias assessment

Using the Cochrane Collaboration ROB (risk of bias) tool, we assessed the methodological quality of the study (35). Every study evaluated ROB in seven domains, categorizing it as high, unclear, or low. Low ROB studies were defined as three or less as unclear risk and none as high risk. Moderate ROB studies were defined as none rated as high risk but four or more were rated as unclear risk, or one was rated as high risk. All other studies have identified higher ROB studies.

### Measures of treatment effect

#### Data synthesis

Continuous and dichotomous variables were analyzed using mean difference (MD) and odds ratio (OR), respectively. An NMA with random effects was used to estimate effect sizes using MDs or ORs with a 95% credible interval (CrI). Continuous and dichotomous outcomes were used for normal and binomial likelihoods, respectively. Model convergence was satisfactory when the potential scale reduction factor approached 1.0 (36). The treatments were evaluated and ranked according to the surface area under the cumulative ranking curve (SUCRA) (37).

#### Assessment of heterogeneity

Statistically significant heterogeneity was *I*^2^ greater than 50%, and we discussed the sources of heterogeneity (38–40).

#### Assessment of inconsistency

Node splitting and design-by-treatment tests were used to assess inconsistencies (39, 41). A *p*-value less than 0.05 was considered an inconsistency between the indirect and direct comparisons.

#### Assessment of transitivity

In order to test the transitivity assumption of NMA, the distribution of clinical variables was compared (37, 42).

#### Subgroup analysis

Subgroup analyses for the primary outcome were evaluated using population, duration of analgesia, and quality of the study. The patients were divided into postoperative critical and general critical groups. The duration of analgesia was divided into the short-term (≤72 h) and long-term (>72 h).

#### Sensitivity analysis

Sensitivity analysis was evaluated through studies quality and studies without publication bias datasets.

#### Quality assessment

GRADE was used to assess the certainty of evidence contributing to the network estimates (high, moderate, low, or very low) (43). Additionally, the comparison-adjusted funnel plots were used to assess publication bias (44, 45).

#### Statistical software

R software, Stata, and Review Manager were used for analysis.

## Results

### Results of the search

**Figure 1:**
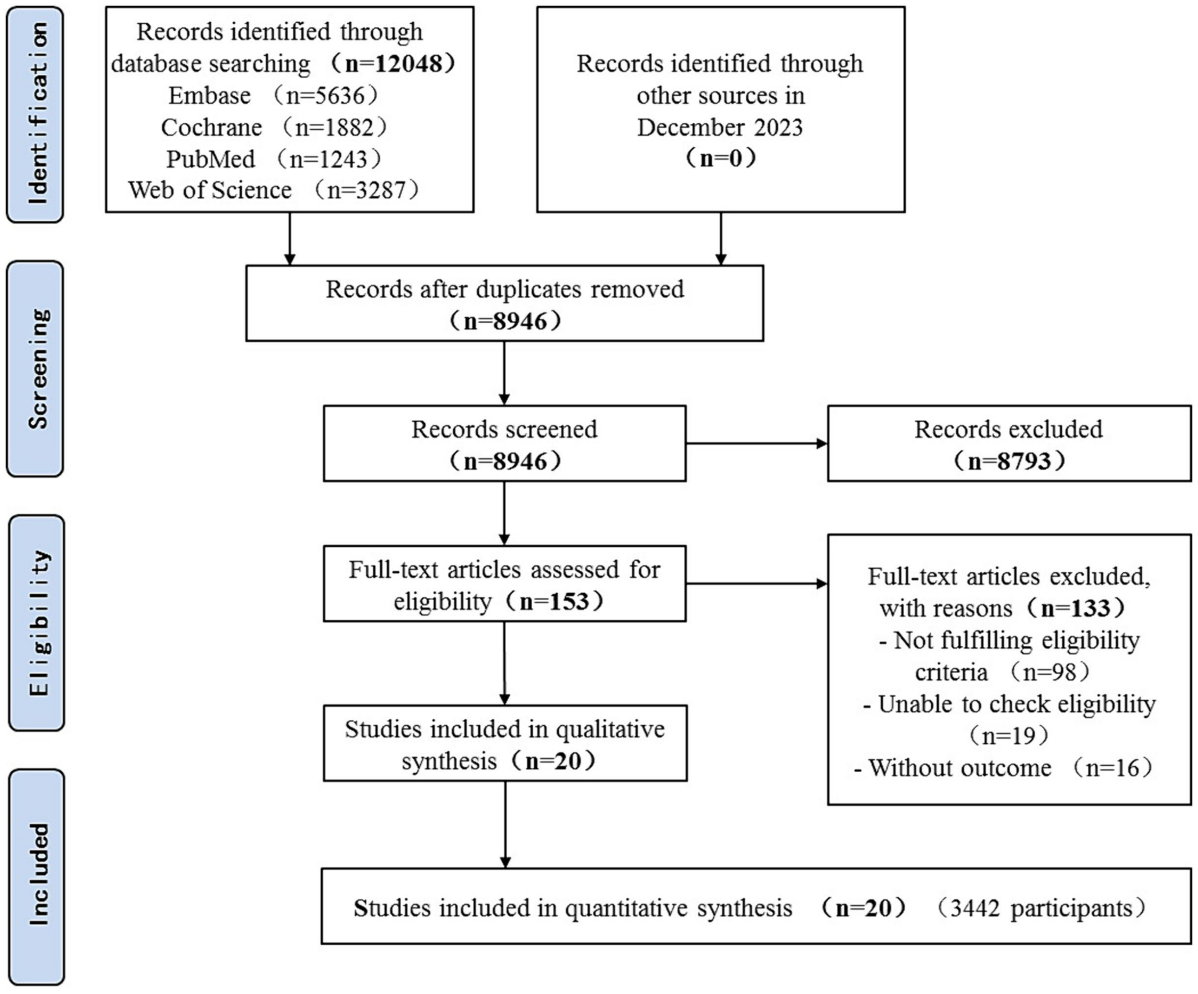
Flow diagram of included studies.

Over 12,048 articles were identified, of which 153 in full-text were potentially eligible for inclusion. In total, 20 RCTs involving 3,442 patients were identified (Figure 1).

### Description of included studies

A total of 20 studies have been published in 12 countries between 1997 and 2023 (25, 29–31, 33, 46–60). There were 14 English articles, 3 Chinese articles, and 1 each in Turkish, French, and Tunisian. A total of nine (45%) trials recruited patients from Asia, seven (35%) trials recruited patients from Europe, two (10%) trials recruited patients from America, and each (5%) trial recruited patients from Oceania and Africa. Study samples ranged from 19 to 681 participants, with an average of 69 (standard deviation [SD] = 84). Participants were 55 years old (SD = 17 years), and 59% were men. Participants in one study (5%) were randomly assigned to three groups, and six (30%) were conducted at different research centers. In total, 14 (70%) studies were double-blind. The most critical patients were postoperative in the ICU, followed by those with brain trauma alone, severe multiple traumas, sepsis, and septic shock. Ten studies involved remifentanil versus fentanyl, 6 studies involved remifentanil versus morphine, and 1 study involved remifentanil versus sufentanil. There were three other studies involving fentanyl and morphine and two studies involving sufentanil versus morphine. Despite this, there have been no studies examining the interactions between sufentanil and morphine. The dose of opioids varied among studies; remifentanil, 0.05–1.0 ug/kg•min; fentanyl, 0.015–2.0 ug/kg•min; morphine, 0.75–2 ug/kg•min; sufentanil, 0.002–0.005 ug/kg•min (Tables 1, 2).

**Table 1:** Description of included studies.

ID	Author	Year	Country	Participants	Design	*N*	Mean age (SD)	Male (%)	Weight (kg)	Height (cm)	Evaluation	Analgesia/Sedation score	Study drug
1	Yamush	1997	America	MV patients in ICU after surgery	MC/DB	72	43.4 (14.9)	26	NR	NR	NR	NR	Remifentanil
						78	44.4 (17.2)	40	NR	NR	NR	NR	Morphine
2	Chinachoti	2002	Thailand	MV patients in ICU with normal renal function or mild renal impairment	MC/DB	74	58.8 (14)	30	71.4 (16)	167.3 (8.6)	SAPS II 25.8 (9.6)	PI/ SAS 1.9 (1.1)/3.5 (1.1)	Remifentanil
						78	59.9 (14.2)	35	71.0 (17.8)	167.2 (8.7)	SAPS II 25.6 (8.5)	PI/ SAS 1.6 (1.1)/3.4 (1.1)	Morphine
3	Dahaba	2004	Austria	MV patients in ICU after orthopedic and general surgery	SC/DB	20	58 (19)	60	69 (17)	NR	SAPS II 24 (7)	NR	Remifentanil
						20	54 (20)	50	76 (16)	NR	SAPS II 22 (4)	NR	Morphine
4	Karabinis	2004	Greece	MV patients in NICU	MC/OP	84	46.8 (16.3)	52	76.5 (12.2)	171.1 (9.1)	GCS 8.4 (2.7)	PI/ SAS 2.1 (1.1)/3.7 (1.5)	Remifentanil
						37	49.6 (16.9)	65	76.5 (12.6)	170.9 (7.4)	GCS 8.8 (2.9)	PI/ SAS 2.1 (1.0)/3.6 (1.2)	Fentanyl
						40	47.3 (20.0)	63	75.2 (12.2)	170.9 (8.5)	GCS 8.6 (2.5)	PI/ SAS 2.1 (1.0)/3.7 (1.5)	Morphine
5	Muellejans	2004	Germany	MV patients in ICU	MC/DB	77	61.5 (13.4)	71	77.2 (12.7)	170.4 (9.1)	SAPS II 28.2 (8.8)	PI/ SAS 1.4/3.2	Remifentanil
						75	58.7 (13.9)	69	74.8 (13.9)	169.6 (9.6)	SAPS II 27.7 (8.8)	PI/ SAS 1.5/3.5	Fentanyl
6	Akinci	2005	Turkey	MV patients in ICU after surgery	SC/DB	22	32 (15)	64	NR	NR	APACHE II 13 (7)	BPS/SAS 5/5	Remifentanil
						22	44 (16)	55	NR	NR	APACHE II 16 (6.75)	BPS/SAS 5/5	Fentanyl
7	Baillard	2005	France	MV patients in ICU	SC/ DB	21	59 (19)	80	66 (12)	NR	NR	NR	Remifentanil
						20	58 (19)	68	70 (12)	NR	NR	NR	Sufentanil
8	Amor	2007	Tunisie	MV patients in ICU with normal renal function or mild renal impairment	SC/DB	9	58 (20)	67	76 (15)	171 (87)	APACHE II 21 (7)	NR	Remifentanil
						10	57 (20)	70	77 (15)	170 (89)	APACHE II 20 (7)	NR	Fentanyl
9	Carrer	2007	Italy	MV patients in ICU after major surgery	SC/DB	50	69 (9)	56	75 (15)	NR	SAPS II 26.1 (7.2)	NR	Remifentanil
						50	69 (10)	51	71 (17)	NR	SAPS II 26.3 (9.5)	NR	Morphine
10	Spies	2010	Germany	MV patients in ICU	MC/DB	28	64 (15)	71	BMI 27 (5)		APACHE II 24 (8)	NR	Remifentanil
						32	63 (12)	84	BMI 26 (4)		APACHE II 26 (9)	NR	Fentanyl
11	Cevik	2011	Turkey	MV patients in ICU	SC/OP	16	50.63 (25.24)	44	65.94 (11.89)	NR	APACHE II 9.56 (3.83)	NR	Remifentanil
						16	51.88 (20.77)	63	70.06 (15.12)	NR	APACHE II 11.94 (6.4)	NR	Fentanyl
12	Oliver	2011	America	MV patients in ICU after cardiopulmonary bypass	SC/DB	38	62 (4)	66	83 (5.25)	173 (3.5)	NR	NR	Fentanyl
						41	63 (4.75)	61	82 (8.25)	175 (4.25)	NR	NR	Morphine
13	Liu	2013	China	MV patients in ICU after tumor operation	SC	30	66.8 (7.8)	33	67.2 (10.8)	NR	APACHE II 21.0 (4.9)	NR	Remifentanil
						30	64.3 (9.3)	27	68.3 (10.9)	NR	APACHE II 20.2 (3.8)	NR	Fentanyl
14	Lee	2014	Korea	MV patients in ICU	MC/OP	49	6 6 (14.5)	67	60.6 (13.4)	162.1 (9.4)	APACHE II 23.4 (8.7)	NR	Remifentanil
						47	66 (15.2)	55	58.1 (10.2)	160.7 (8.9)	APACHE II 21.4 (7.8)	NR	Morphine
15	Yang	2014	China	MV patients in ICU	MC/DB	282	53.6 (19.4)	66	66.6 (10.4)	NR	APACHE II 23.1 (8.7)	FPS/RS 7.1/1.6	Sufentanil
						262	54.6 (20.0)	66	65.3 (11.5)	NR	APACHE II 22.9 (7.5)	NR	Fentanyl
16	Yue	2016	China	MV patients in ICU after major surgery	SC/OP	300	58.3 (10.4)	56	60.2 (5.8)		NR	NR	Sufentanil
						300	59.1 (15.1)	55	59.8 (11.3)		NR	NR	Fentanyl
17	Liu	2017	China	MV patients in ICU after surgery	SC/DB	35	66.11 (11.94)	60	65.29 (17.54)	NR	APACHE II 19.2 (4.19)	BPS/CPOT 4 (0.74)/3 (1.48)	Remifentanil
						35	62 (9.96)	49	67.66 (9.95)	NR	APACHE II 20.20 (5.04)	BPS/CPOT 4 (0.74)/4 (0.74)	Fentanyl
18	Casamento	2021	Australia	MV patients in ICU	MC/OP	344	56.9 (17.9)	63	84.6 (22.4)	NR	APACHE II 16.6 (6.7)	NR	Fentanyl
						337	58.5 (19.9)	62	82.4 (18.5)	NR	APACHE II 17.7 (7.4)	NR	Morphine
19	Doi	2023	Japan	MV patients in ICU	MC/DB	98	68.5 (10.9)	75	BMI 21.54 (3.69)		NR	BPS 3.6 (1.1)	Remifentanil
						98	65.9 (13.2)	80	BMI 22.41 (3.86)		NR	BPS 3.5 (1.1)	Fentanyl
20	Li	2023	China	MV patients in ICU	MC/DB	69	59.3 (16.3)	61	66.4 (16.3)	166.3 (8.5)	APACHE II 12.5 (5.56)	CPOT/RASS 0.4 (0.74)/−1.4 (1.11)	Remifentanil
						68	59.4 (18.2)	72	64.1 (12.6)	165.0 (7.4)	APACHE II 13.0 (6.67)	CPOT/RASS 0.4 (0.74)/−0.3 (0.74)	Fentanyl

APACHE II, acute physiology and chronic health evaluation; BMI, body mass index; BPS, behavioral pain scale; CPOT, critical care pain observation tool; DB, double-blind; GCS, Glasgow coma scale; MC, multi-center; MV, mechanical ventilation; NICU, neurological intensive care unit; NR, not reported; OP, open study; PI, pain intensity score; RASS, Richmond agitation-sedation scale; RCT, randomized controlled trials; SC, single-center; SD, mean deviations; SAPS II, simplified acute physiology score; SAS, sedation agitation score.

**Table 2:** Description of included studies.

ID	Author	Participants	Post-surgical patients (%)	Details of study drug	Supplement analgesic/sedative	Aim	Outcomes
1	Yamush	MV patients in ICU after surgery	100	Remifentanil: 0.025 ug/kg/min	No supplement analgesic/sedative	PI≤1	Duration of extubation, ICU LOS, and bradypnea
			100	Morphine: 2 mg bolus (every 5 min)			
2	Chinachoti	Post-surgical and medical ICU patients requiring MV for 12–72 h	98.6	Remifentanil: 0.15–1 ug/kg/min	Two groups were given midazolam 0.03–0.2 mg/kg/h when the dose of the study drug reached the midazolam “trigger dose”	PI≤2 and SAS = 4	Duration of MV, duration of extubation, ICU mortality, efficacy, safety, and bradypnea
			98.7	Morphine: 0.75–5 ug/kg/min With bolus 10ug/kg (over 60s)			
3	Dahaba	MV patients in ICU after orthopedic and general surgery	100	Remifentanil: 0.15–0.2 ug/kg/min	Two groups were given midazolam a 30 ug/kg bolus and 0.5 ug/kg/min when the dose of the study drug reached the midazolam “trigger dose.” Increased 0.125 ug/kg/min accompanied with a bolus of 15 ug/kg or decreased by 0.125 ug/kg/min	PI≤2 and SAS = 4	Duration of MV, duration of extubation, ICU LOS, ICU mortality, efficacy, safety, and hypotension
			98.7	Morphine: 0.75–5 ug/kg/min With bolus 25 ug/kg (over 60s)			
4	Karabinis	MV patients in NICU	37	Remifentanil: 0.15-1ug/kg/min	From the first day to the third day, the three groups were given propofol a 0.5 mg/kg bolus, and 0.5 ug/kg/h when the dose of the study drug reached the propofol “trigger dose.” Starting on the fourth day, all patients changed to midazolam infusion (0.01–0.5 mg/kg bolus and 0.03–0.3 ug/kg/h)	PI≤2 and SAS < 4	Duration of MV, duration of extubation, ICU LOS, ICU mortality, efficacy, safety, and bradycardia
			49	Fentanyl: follow the clinical practice routines of each investigating site			
			25	Morphine: follow the clinical practice routines of each investigating site			
5	Muellejans	MV patients in ICU	92	Remifentanil: 0.15–0.2 ug/kg/min	Two groups were given propofol a 0.5 mg/kg bolus and 0.5 ug/kg/h when the dose of the study drug reached the propofol “trigger dose.” Increased 0.125 mg/kg/h accompanied with a bolus of 0.25 mg /kg or decreased by 0.125 mg/kg/h	PI≤2 and SAS = 4	Duration of MV, duration of extubation, ICU LOS, efficacy, safety, hypotension, and bradycardia
			95	Fentanyl: 1 ug/kg bolus and 1.5–2 ug/kg/h			
6	Akinci	MV patients in ICU after surgery	100	Remifentanil: 0.1 ug/kg/min	Morphine as rescue treatment for two groups	BPS = 3 and SAS = 3	Duration of extubation, hypotension, and bradypnea
			100	Fentanyl: 0.025 ug/kg/min			
7	Baillard	MV patients in ICU	29	Remifentanil: 0.17 ug/kg/min	Two groups were given midazolam 0.1 mg/kg/h	RS 2–4	Duration of extubation, ICU LOS, ICU mortality, and efficacy
			20	Sufentanil: 0.002 ug/kg/min			
8	Amor	MV patients in ICU with normal renal function or mild renal impairment	0	Remifentanil: 6 ug/kg/h, titrated up by increment of 100 ug/h	Two groups were given midazolam 0.1 mg/kg/h	RS 3–4	Duration of MV, duration of extubation, and ICU LOS
			0	Fentanyl: 1.5 ug/kg/h, titrated up with an increment of 25 ug/h			
9	Carrer	MV patients in ICU after major surgery	100	Remifentanil:0.1 ug/kg/min stepwise variations by ±25% and boluses allowed (0.025 μg/kg in 30 s)	Two groups were given morphine 0.24 mg/kg/h while in patients aged 75 years 0.12 μg/kg/min	RS 2–3 and NRS < 3	Duration of MV, ICU LOS, ICU mortality, safety, hypotension, bradycardia, and bradypnea
			100	Morphine:0.48ug/kg/min stepwise variations by ±25%, and boluses allowed (0.1 mg/kg in 30 s)			
10	Spies	MV patients in ICU	92	Remifentanil: 0.1–0.4 ug/kg/min (IBW)	Two groups were given morphine for rescue pain and were given midazolam 0.01–0.18 mg/kg/h, propofol 4 mg/kg/h for sedation	VAS ≤3 and/or BPS ≤6	Duration of MV and ICU LOS
			97	Fentanyl: 0.02–0.08 ug/kg/min (IBW)			
11	Cevik	MV patients in ICU	88	Remifentanil:0.05 ug/kg/min (initial dose) Increased 0.05 ug/kg/min	Two groups were given midazolam at an initial dose of 0.03 mg/kg/h	RS ≤3	Duration of MV, ICU LOS, safety, hypotension, and bradycardia
			88	Fentanyl:0.015 ug/kg/min (initial dose) Increased 0.01 ug/kg/min			
12	Oliver	MV patients in ICU after cardiopulmonary bypass	100	Fentanyl: 0.5 ug/kg/h	Two groups were given propofol 25 ug/kg/min	VAS ≤3 and RS >3	Duration of extubation and ICU LOS
			100	Morphine boluses			
13	Liu	MV patients in ICU after tumor operation	100	Remifentanil:0.05–0.1 ug/kg/min	Two groups were given propofol 0.5 mg/kg/h when the dose of the study drug reached the propofol “trigger dose”	FPS ≤ 2 RS 2–3	Duration of MV, ICU LOS, safety, hypotension, bradycardia, and bradypnea
			100	Fentanyl: 0.5–1 ug/kg/h and 0.7–1.5 ug/kg bolus when necessary			
14	Lee	MV patients in ICU	9	Remifentanil: 0.1–0.2 ug/kg/min	Midazolam as rescue treatment for two groups	NR	Duration of extubation
			12	Morphine: 0.8–35 mg/h			
15	Yang	MV patients in ICU	0	Sufentanil:≤0.3 ug/kg/h	Two groups were given midazolam when the dose of the study drug reached the midazolam “trigger dose”	FPS ≤ 2 or RS = 3	Safety, hypotension, bradycardia, and bradypnea
			0	Fentanyl: ≤2 g/kg/h			
16	Yue	MV patients in ICU after major surgery	100	Sufentanil: 5 ug/h	Two groups were given propofol 1 mg/kg bolus as rescue treatment	Prince-Henry 0–1 RASS -1 ~ 0	Safety, hypotension, bradycardia, and bradypnea
			100	Fentanyl: 50 ug/h			
17	Liu	MV patients in ICU after surgery	100	Remifentanil: 1 ug/kg/h	Three groups were given midazolam infusion (0.05 mg/kg bolus and 0.02–0.1 ug/kg/h)	RASS-3 ~ −1	Duration of MV, duration of extubation, and ICU LOS
			100	Fentanyl: 50 ug/h			
18	Casamento	MV patients in ICU	35.8	NR	NR	-2 ≤ RASS≤1	Duration of MV, ICU LOS, ICU, and mortality
			34.4	NR			
19	Doi	MV patients in ICU	100	Remifentanil: 1.5 ug/kg/h (Initial dose) Increased 1.5 ug/kg/h	Fentanyl was administered as a rescue analgesic	BPS ≤ 5 or NRS ≤ 3	Duration of MV, duration of extubation, ICU mortality, efficacy, safety, hypotension, and bradypnea
			100	Fentanyl: 0.1 ug/kg/h (Initial dose) Increased 0.1 ug/kg/h			
20	Li	MV patients in ICU	70	Remifentanil: 1.5 ug/kg/h (Initial dose) Increased 1.5 ug/kg/h	Two groups were given propofol 0.5 mg/kg/h when the dose of the study drug reached the propofol “trigger dose”	CPOT≤2 −2 ≤ RASS≤1	Duration of MV, duration of extubation, ICU LOS, ICU mortality, efficacy, safety, hypotension, and bradycardia
			70	Fentanyl: 1 ug/kg bolus and 0.25 ug/kg/h (Initial dose) Increased 0.25 ug/kg/h			

BPS, behavioral pain scale; CPOT, critical care pain observation tool; FPS, facial pain scale; GCS, Glasgow coma scale; IBW, ideal body weight; MV, mechanical ventilation; NR, not reported; NRS, numerical rate score; PI, pain intensity score; RASS, Richmond agitation sedation scale; RS, Ramsey scale; SAS, sedation agitation score; VAS, visual analog scale.

A total of 13 studies reported the duration of MV, 13 studies reported the duration of extubation, 14 reported ICU LOS, and 8 reported ICU mortality. In total, 7 studies reported efficacy, 11 reported safety, 10 reported drug-related hypotension, 8 reported drug-related bradycardia, and 8 reported drug-related bradypnea (Table 3).

**Table 3:** Reported clinical outcomes of included studies.

ID	Study drug	Duration of MV (hours)	Duration of extubation (hours)	ICU LOS (days)	ICU Mortality (n/N)	Efficacy (%/hours)	Safety (n/N)	Hypotension (n/N)	Bradycardia (n/N)	Bradypnea (n/N)
1	Remifentanil	NR	0.10 (0.10)	0.11 (0.11)	NR	NR	NR	NR	NR	10/72
	Morphine	NR	0.14 (0.20)	0.10 (0.11)	NR	NR	NR	NR	NR	5/78
2	Remifentanil	17.20 (10.51)	1.50 (1.90)	NR	2/106	94.5 (24.28)	23/106	NR	NR	4/106
	Morphine	16.90 (8.65)	2.50 (4.00)	NR	1/83	93.9 (23.88)	13/83	NR	NR	10/83
3	Remifentanil	14.38 (2.85)	0.28 (0.10)	1.46 (0.19)	0/20	78.3 (6.2)	8/20	1/20	NR	NR
	Morphine	19.32 (3.46)	1.22 (0.12)	2.54 (0.39)	0/20	66.5 (8.5)	6/20	0/20	NR	NR
4	Remifentanil	25.83 (24.56)	1.0 (24.30)	2.85 (1.77)	4/84	95.6 (21.25)	21/84	NR	1/84	NR
	Fentanyl	24.76 (14.05)	0.68 (1.40)	2.79 (1.41)	0/37	98.1 (3.25)	3/37	NR	0/37	NR
	Morphine	38.97 (26.65)	1.93 (24.05)	3.61 (1.69)	2/40	99.0 (25)	4/40	NR	0/40	NR
5	Remifentanil	14.7 (19.61)	1.00 (5.25)	1.70 (1.68)	NR	89.5 (13.7)	26/115	19/115	2/115	NR
	Fentanyl	15.3 (18.79)	1.10 (1.125)	1.65 (1.69)	NR	89.3 (16.88)	14/81	8/81	3/81	NR
6	Remifentanil	NR	0.10 (3.23)	NR	NR	NR	NR	10/22	NR	3/22
	Fentanyl	NR	0.10 (7.05)	NR	NR	NR	NR	11/22	NR	10/22
7	Remifentanil	NR	22 (30.37)	26.00 (27.41)	12/21	89.0 (46.13)	NR	NR	NR	NR
	Sufentanil	NR	96 (70.37)	19.00 (17.04)	12/20	89.0 (44.87)	NR	NR	NR	NR
8	Remifentanil	132 (79)	24.67 (16.34)	15.00 (13.00)	NR	NR	NR	NR	NR	NR
	Fentanyl	129 (66)	48 (21.33)	17.00 (11.00)	NR	NR	NR	NR	NR	NR
9	Remifentanil	17 (6)	NR	2.30 (2.30)	1/50	NR	9/50	0/50	0/50	1/50
	Morphine	18 (4)	NR	2.30 (2.50)	1/50	NR	6/50	0/50	0/50	8/50
10	Remifentanil	136 (218.6)	NR	23.00 (34.83)	NR	NR	NR	NR	NR	NR
	Fentanyl	162 (255.4)	NR	26.00 (34.83)	NR	NR	NR	NR	NR	NR
11	Remifentanil	45.75 (74.71)	NR	8.70 (9.96)	NR	NR	7/16	5/16	2/16	NR
	Fentanyl	45.75 (47.13)	NR	9.88 (6.66)	NR	NR	5/16	5/16	0/16	NR
12	Fentanyl	NR	4.67 (0.50)	0.97 (0.33)	NR	NR	NR	NR	NR	NR
	Morphine	NR	4.73 (0.54)	0.96 (0.02)	NR	NR	NR	NR	NR	NR
13	Remifentanil	73.6 (26.7)	NR	5.25 (1.55)	NR	NR	13/30	8/30	3/30	0/30
	Fentanyl	94.9 (37.3)	NR	6.28 (2.12)	NR	NR	5/30	2/30	1/30	0/30
14	Remifentanil	NR	90 (89)	NR	NR	NR	NR	NR	NR	NR
	Morphine	NR	144 (176)	NR	NR	NR	NR	NR	NR	NR
15	Sufentanil	NR	NR	NR	NR	NR	34/282	9/282	6/282	12/282
	Fentanyl	NR	NR	NR	NR	NR	39/262	18/262	5/262	15/262
16	Sufentanil	NR	NR	NR	NR	NR	11/300	0/300	0/300	11/300
	Fentanyl	NR	NR	NR	NR	NR	21/300	0/300	0/300	21/300
17	Remifentanil	102 (65.93)	12 (17.22)	6.00 (3.70)	NR	NR	NR	NR	NR	NR
	Fentanyl	126 (139.3)	18 (37.78)	7.00 (6.01)	NR	NR	NR	NR	NR	NR
18	Fentanyl	61.84 (79.23)	NR	4.38 (4.37)	34/344	NR	NR	NR	NR	NR
	Morphine	72.74 (88.90)	NR	4.96 (4.67)	46/337	NR	NR	NR	NR	NR
19	Remifentanil	7.03 (11.99)	1.68 (4.31)	NR	0/92	99.16 (2.60)	12/92	3/92	NR	2/92
	Fentanyl	6.88 (12.95)	1.17 (2.68)	NR	0/90	98.50 (3.44)	15/90	3/90	NR	0/90
20	Remifentanil	26.11 (21.33)	0.94 (0.72)	2.45 (1.17)	1/69	98.75 (2.13)	24/69	47/69	14/69	NR
	Fentanyl	25.34 (20.22)	1.20 (1.27)	2.29 (0.68)	1/68	98.50 (2.76)	22/68	47/68	11/68	NR

Continuous variables are represented by means and standard deviations.LOS, Length of stay; MV, mechanical ventilation; NR, not reported.

### ROB in included studies

**Figure 2:**
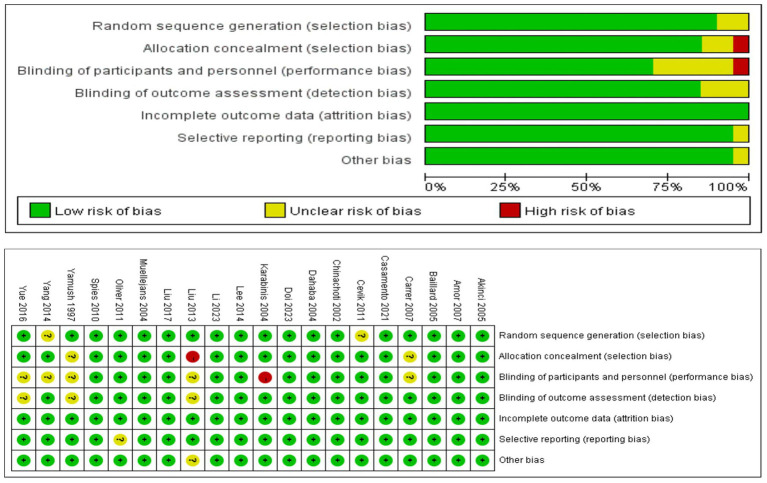
Risk of bias.

In summary (Figure 2), 17 (85%) of the 20 trials were rated as having low ROB, and 3 (20%) as having moderate ROB.

### Effects of interventions

#### Primary outcome (duration of MV)

**Figure 3:**
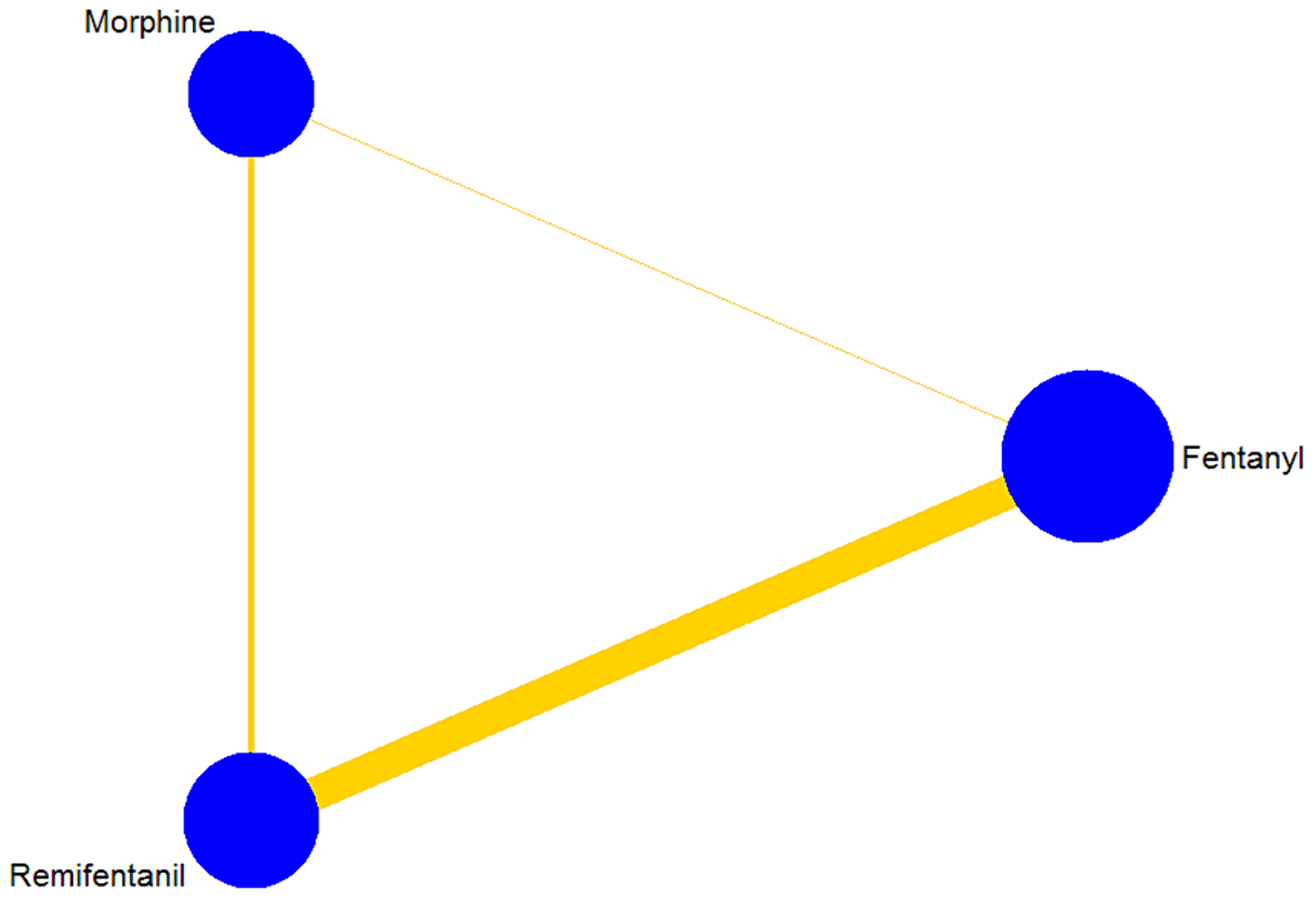
Network plot of all intervention comparisons for the duration of mechanical ventilation. The node size corresponds to the total number of participants in this study’s treatments. The comparable treatments are linked with a line. The colors and thickness of the line correspond to the quality and standard error of trials that study this comparison, respectively. Low risk of bias is green, moderate risk of bias is yellow.

An analysis of 13 studies, including 1860 patients, was conducted to determine the duration of MV. There were 9, 4, and 2 trial arms involving direct comparisons of remifentanil and fentanyl, remifentanil and morphine, and morphine and fentanyl, respectively. None of the studies on sufentanil were included. All the Bayesian parameters converged well. Figure 3 displays a network of eligible comparisons for the MV duration.

The results of the NMA are shown in Table 4 for the duration of MV. Compared with remifentanil, when fentanyl and morphine were administered to analgesia, the duration of MV was not significantly prolonged (MD -0.16; 95% CrI: −4.75 to 5.63) and (MD 3.84; −0.29 to 10.68), respectively. The differences between the three opioids were not significant. The SUCRA results showed that the best possible interventions for achieving the shortest duration of MV were remifentanil (46.0%), fentanyl (52.2%), and morphine (1.8%) (Supplementary Figure S8.1). However, we cannot conclude from the above results that fentanyl is the best regimen to shorten the duration of MV among the three opioids.

**Table 4:** Results from pairwise meta-analyses and network meta-analyses on mechanical ventilation.

Fentanyl	13.14 (2.54, 23.17)	−0.95 (−6.23, 3.14)
−4.09 (−11.38, 1.94)	Morphine	−2.60 (−7.72, 1.41)
−0.16 (−4.76, 5.65)	3.85 (−0.26, 10.74)	Remifentanil

Data are the MDs (95% CrI) in the column-defining treatment compared with the row-defining treatment. With treatment as the boundary, the lower left part of the table is the result of network meta-analyses, and the upper right part of the table is the result of pairwise meta-analyses. For network meta-analyses, MDs lower than 0 favor the column-defining treatment: e.g., column 1 vs. row 3 in the lower left part of the table (Fentanyl vs. Remifentanil) is the result of network meta-analyses (MDs − 0.16 95% CrI -4.76 to 5.65). For pairwise meta-analyses, MDs higher than 0 favor the row-defining treatment: e.g., column 3 vs. row 1 in the upper right part of the table (Remifentanil vs. Fentanyl) is the result of pairwise meta-analyses (MDs − 0.95 95% CrI -6.23 to 3.14). MDs, mean differences; CrI, credible interval.

#### Secondary outcomes

**Figure 4:**
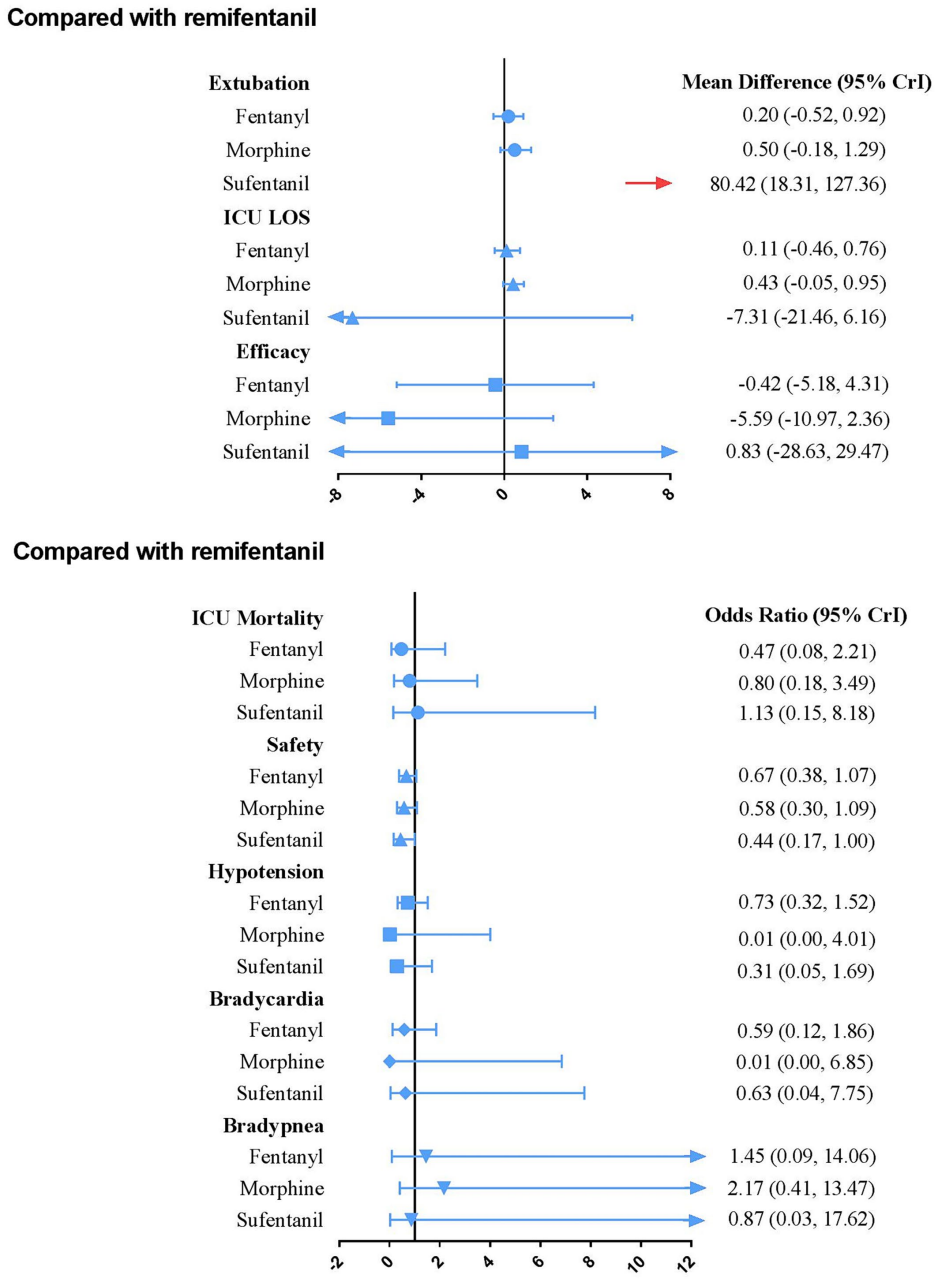
Forest plot for each active intervention versus remifentanil on secondary outcomes estimates are presented as MD (mean difference) or odds ratios (OR) and 95% CrI. OR < 1 favor the treatment. MD < 0 favor the treatment. CrI, credible interval; LOS, Length of stay.

Figure 4 presents the results of secondary outcomes. Compared with remifentanil, sufentanil can prolong the duration of extubation (MD 80.42; 95% CrI 18.31–127.36). No regimen significantly improved ICU LOS, efficacy, safety, and other secondary outcomes. The SUCRA ranking curve showed that remifentanil ranked first for shortening the extubation duration and reducing the occurrence of drug-related bradypnea. Fentanyl ranked first for ICU mortality. Moreover, morphine ranked first for efficacy, reducing the occurrence of drug-related hypotension and bradycardia. Furthermore, sufentanil ranked first for ICU-LOS and safety (Supplementary File 8).

### Direct meta-analysis

A pairwise analysis of the duration of MV is presented in Table 4.

#### Heterogeneity, inconsistency, and transitivity

In terms of MV duration (I^2^ = 68.70%) and ICU LOS (I^2^ = 99.87%), there was moderate-to-high global heterogeneity (Supplementary File 4).

No global inconsistency was observed in any of the outcomes (Supplementary Table S4.2). When the node-splitting model was compared indirectly and directly, there was no evidence of inconsistency.

Most comparisons had similar mean ages in the assessment of transitivity (Supplementary File 5).

#### Subgroup analyses and sensitivity analyses for the duration of MV

Compared with remifentanil, when morphine was administered as analgesia, the duration of MV was significantly prolonged (MD 12.53; 95% CrI: 2.34 to 22.59). The three opioids had similar effects on shortening the duration of MV in each subgroup of patients, regardless of their patient population, duration of analgesia, and study quality (Table 5). In addition, heterogeneity and consistency were not statistically significant among the subgroups.

**Table 5:** Subgroup analyses for the duration of mechanical ventilation in different populations.

Treatment	Overall patients		Postoperative ICU patients		Mixed ICU patients		Analgesia is greater than 72 h		Analgesia is less than 72 h		High quality studies only	
	MDs (95% CrI)	Rank	MDs (95% CrI)	Rank	MDs (95% CrI)	Rank	MDs (95% CrI)	Rank	MDs (95% CrI)	Rank	MDs (95% CrI)	Rank
Fentanyl	−0.16 (−4.75, 5.63)	1	5.44 (−5.37, 23.44)	3	−0.27 (−6.39, 5.78)	1	8.41 (−9.80, 30.97)	2	−1.68 (−8.17, 4.90)	1	−0.62 (−5.62, 4.09)	1
Morphine	3.84 (−0.29, 10.68)	3	1.91 (−9.96, 13.62)	2	**12.53 (2.34, 22.59)**	3	19.34 (−17.40, 61.27)-	3	3.22 (−1.19, 9.66)	3	2.48 (−1.47, 7.19)	3
Remifentanil	Reference	2	Reference	1	Reference	2	Reference	1	Reference	2	Reference	2
Number of studies	13		8		5		6		7		11	
Participants	1860		710		1,150		1,086		774		1,666	

Bold values are compared with remifentanil, when morphine was administeredas analgesia, the duration of MV was significantly prolonged.

The sensitivity analysis did not change substantially (Supplementary File 9).

#### GRADE assessments

Except for the extubation duration, no publication bias was found (Supplementary File 6). The degree of certainty about shortening MV time was variable (Supplementary Table S7.1). For comparisons involving fentanyl, morphine, and remifentanil, it was low, whereas, for comparisons involving morphine and remifentanil, it was very low. The GRADE of ranking of treatment was very low. The GRADE was raised to at least moderate when subgroup analysis was performed. Table 6 and Supplementary File 7 presents details of GRADE.

**Table 6:** Result of GRADE for primary outcome.

	Nature of the evidence	Study limitations	Imprecision	Inconsistency	Indirectness	Publication bias	Confidence	Downgrading due to
A vs. B	Mixed estimated	No downgrade	Downgrade because point estimate <1.0 but upper limit >1.25	Downgrade because pair heterogeneity *I*^2^ = 81.2%	No downgrade	No downgrade	LOW	Imprecision Inconsistency
A vs. C	Mixed estimated	No downgrade	Downgrade because point estimate >1.0 but lower limit<0.80	No downgrade	No downgrade	Downgrade because publication bias	LOW	Imprecision Publication bias
B vs. C	Mixed estimated	Downgrade because >70% contribution from moderate ROB comparisons	Downgrade because point estimate >1.0 but lower limit<0.80	Downgrade because pair heterogeneity *I*^2^ = 85.1%	No downgrade	Downgrade because publication bias	VERY LOW	Study limitations Imprecision Inconsistency Publication bias
Ranking of treatments		No downgrade	Downgrade because similar distributions of ranks	Downgrade because global heterogeneity *I*^2^ = 67.70%	No downgrade	Downgrade because publication bias	VERY LOW	Imprecision Inconsistency Publication bias

A, Fentanyl; B, Morphine; C, Remifentanil.

## Discussion

### Main results summary

This study was conducted to investigate the effect of analgesic regimens using remifentanil, morphine, and fentanyl on the duration of MV. It was concluded that remifentanil did not significantly shorten the duration of MV in mechanically ventilated patients compared to morphine or fentanyl. This finding was supported by sensitivity and subgroup analyses. In addition, the SUCRA ranking curve indicated that fentanyl ranked first among the three opioids for shortening the duration of MV, but the difference was not statistically significant.

### Applicability of evidence

Remifentanil did not reduce the duration of MV, which is consistent with the previous conclusion that all opioids administered intravenously appear to exhibit a similar duration of MV when titrated to similar pain intensity endpoints (5). However, the pharmacokinetics of remifentanil is not similar to those of morphine and fentanyl. The results were interpreted carefully for the following reasons: First, elimination independent of renal function seems to make remifentanil more effective in patients with renal impairment (20). Amor and Chinachoti’s study focused on patients with mild renal impairment, although not suggested remifentanil can shorten the duration of MV, they indicated remifentanil was associated with shorter the duration of weaning (47, 50). In Chinachoti et al.’s study, it should be noted that twice the amount of midazolam in the morphine group may have reduced morphine-related side effects (47). Second, a prolonged infusion did little to affect the context-sensitive half-life of remifentanil. Remifentanil shortened the duration of MV by at least 24 h when analgesia was > 5 days (29, 33, 57). Although the difference was not statistically significant, it is important to avoid ventilator-associated pneumonia, improve ICU outcomes, and reduce costs (23, 61). This suggests that remifentanil is the most suitable treatment for mechanically ventilated patients undergoing long-term analgesia (28). Third, as a result of remifentanil’s rapid onset and offset action, it permitted a significantly quicker and more predictable awakening when it came to performing neurological assessment (31). Thus, although the reduced duration between remifentanil and either of the comparator opioids was less than 1 h, remifentanil may be more meaningful for these patients (31, 62). Fourth, the agents and sedation protocols used differed between studies. Seven studies used midazolam as an adjuvant sedative, and the other three used propofol as an adjuvant sedative. It was more difficult to estimate the effect of opioids when sedatives and analgesics were combined. Finally, heterogeneity and publication bias were the main reasons for the reduction in the GRADE scores. Therefore, these factors weaken the inference drawn from the current findings. Larger, well-powered, and more definitive clinical trials based on different populations are urgently needed to avoid such biases.

### Analysis of secondary outcomes

In terms of extubation duration, sufentanil showed a prolonged effect compared with remifentanil. However, these findings were inconclusive. We need to note that the CrI was too wide because this result was only determined in one study that enrolled 41 patients on MV and was stopped after an interim analysis (48). Therefore, caution should be exercised when interpreting the impact of sufentanil, and it is imperative to conduct future large RCTs to validate these clinical results. Neither of the four opioid medications significantly differed in ICU-LOS, ICU mortality, efficacy, safety, or drug-related adverse events. It can be interpreted for two reasons. First, all available IV opioids were equally effective when titrated to similar pain intensity end points (5). Second, the frequent reassessment of pain and careful titration of analgesic interventions were helpful in preventing negative sequelae due to excessive or inadequate analgesic therapy (63).

### Strengths of this NMA

This study has several strengths. First, this is the first NMA to assess the effectiveness of IV opioid μ-receptor analgesics to shorten the duration of MV in mechanically ventilated patients. Second, it was the most updated evaluation of IV opioid μ-receptor analgesics for patients on MV. A structured search strategy retrieved all identified studies. Third, several relevant clinical outcomes were examined in a heterogeneous population. Fourth, we focused on the co-interventions of sedatives and included only studies that employed the same strategies for sedation. Finally, this study focused on a wide range of clinical outcomes.

### Limitations of this NMA

There are still several limitations in drawing strong treatment inferences. First, several studies did not provide accurate study criteria, such as mode of MV, weaning, and extubation. It is difficult to make these definitions consistent. In addition, the varying opioid doses, sedative types, length of administration, and consumption in different studies weakened any possible recommendations and conclusions. Second, because of the inconsistency in adjuvant sedatives, fewer eligible studies were included and subgroup analyses could not be performed. Therefore, we downgraded the GRADE score. Third, many comparisons had low-level evidence. Mainly because of a wide 95% CrI, possibly implying a small number of studies. Finally, European and Asian countries accounted for 80% of all studies.

## Conclusion

This study provides evidence that remifentanil, compared with fentanyl and morphine, does not shorten the duration of MV in ICU patients. Clinicians should carefully titrate the analgesia of mechanically ventilated patients to prevent a potentially prolonged duration of MV. As such, based on current data, no final recommendations or conclusions can be made. Further large-scale multicenter RCTs according to the characteristics of different populations, especially organ failure patients and long-term analgesic patients, are needed to clarify the most appropriate analgesics, dosages, duration of infusion, and strategies of analgesia.

## Data availability statement

The original contributions presented in the study are included in the article/Supplementary material, further inquiries can be directed to the corresponding authors.

## Author contributions

FL: Data curation, Formal analysis, Investigation, Methodology, Project administration, Writing – original draft. SQ: Data curation, Formal analysis, Investigation, Methodology, Writing – review & editing. ChL: Data curation, Formal analysis, Investigation, Writing – review & editing. XC: Data curation, Investigation, Project administration, Writing – review & editing. ZD: Data curation, Formal analysis, Investigation, Project administration, Writing – review & editing. CoL: Data curation, Formal analysis, Funding acquisition, Investigation, Methodology, Project administration, Resources, Supervision, Validation, Writing – review & editing, Writing – original draft.

## Footnotes

^1^ http://www.controlled-trials.com/isrctn/

^2^ http://www.who.int/ictrp/en/

^3^ www.chictr.org

## Glossary

AE: Adverse events
APACHE II: Acute Physiology and Chronic Health Evaluation II
CrI: Credible interval
DB: Double-blind
GCS: Glasgow coma scale
GRADE: Grades of Recommendation, Assessment, Development and Evaluation
ICU: Intensive care unit
LOS: Length of stay
MC: Multicenter
MD: Mean difference
MV: Mechanical ventilation
NMA: Network meta-analysis
NR: Not reported
OP: Open study
OR: Odds ratio
PRISMA: Preferred Reporting Items for Systematic Review and Meta-analysis
PROSPERO: Prospective register of systematic reviews
RCTs: Randomized controlled trial studies
SAPS: Simplified acute physiology score
SB: Single-blind
SC: Single-center
SD: Standard deviation
SUCRA: Surface under the cumulative ranking curve
