# Supplementary material for: ICU patients receiving remifentanil do not experience reduced duration of mechanical ventilation: a systematic review of randomized controlled trials and network meta-analyses based on Bayesian theories
Source: Front Med (Lausanne). 2024 Aug 7;11:1370481. doi: 10.3389/fmed.2024.1370481 (PMC11342801; doi:10.3389/fmed.2024.1370481)
Supplement: Supplementary file 1 [file Data_Sheet_1.DOC]

# Additional file 1

**Search strategy for pubmed**

1. **("Critical Illness"[Mesh]) OR ((((Critical Illnesses[Title/Abstract]) OR (Illness, Critical[Title/Abstract])) OR (Illnesses, Critical[Title/Abstract])) OR (Critically Ill[Title/Abstract]))**
2. ("Critical Care"[Mesh]) OR ((((((Care, Critical[Title/Abstract]) OR (Intensive Care[Title/Abstract])) OR (Care, Intensive[Title/Abstract])) OR (Surgical Intensive Care[Title/Abstract])) OR (Care, Surgical Intensive[Title/Abstract])) OR (Intensive Care, Surgical[Title/Abstract]))
3. ("Intensive Care Units"[Mesh]) OR ((((Intensive Care Unit[Title/Abstract]) OR (Unit, Intensive Care[Title/Abstract])) OR (ICU Intensive Care Units[Title/Abstract])) OR (ICU[Title/Abstract]))
4. ("Burn Units"[Mesh]) OR (((((((Burn Unit[Title/Abstract]) OR (Unit, Burn[Title/Abstract])) OR (Units, Burn[Title/Abstract])) OR (Burn Centers[Title/Abstract])) OR (Burn Center[Title/Abstract])) OR (Center, Burn[Title/Abstract])) OR (Centers, Burn[Title/Abstract]))
5. ("Coronary Care Units"[Mesh]) OR (((((Care Unit, Coronary[Title/Abstract]) OR (Care Units, Coronary[Title/Abstract])) OR (Coronary Care Unit[Title/Abstract])) OR (Unit, Coronary Care[Title/Abstract])) OR (Units, Coronary Care[Title/Abstract]))
6. ("Respiratory Care Units"[Mesh]) OR (((((Care Unit, Respiratory[Title/Abstract]) OR (Care Units, Respiratory[Title/Abstract])) OR (Respiratory Care Unit[Title/Abstract])) OR (Unit, Respiratory Care[Title/Abstract])) OR (Units, Respiratory Care[Title/Abstract]))
7. ("Postoperative Care"[Mesh]) OR (((((Care, Postoperative[Title/Abstract]) OR (Postoperative Procedures[Title/Abstract])) OR (Postoperative Procedure[Title/Abstract])) OR (Procedure, Postoperative[Title/Abstract])) OR (Procedures, Postoperative[Title/Abstract]))
8. ((((((((((ICUs[Title/Abstract]) OR (NICU[Title/Abstract])) OR (NICUs[Title/Abstract])) OR (PICU[Title/Abstract])) OR (PICUs[Title/Abstract])) OR (SICU[Title/Abstract])) OR (SICUs[Title/Abstract])) OR (CCU[Title/Abstract])) OR (CCUs[Title/Abstract])) OR (EICU[Title/Abstract])) OR (EICUs[Title/Abstract])
9. **or/1-8**
10. ("Respiration, Artificial"[Mesh]) OR ((((((((((Artificial Respiration[Title/Abstract]) OR (Artificial Respirations[Title/Abstract])) OR (Respirations, Artificial[Title/Abstract])) OR (Ventilation, Mechanical[Title/Abstract])) OR (Mechanical Ventilations[Title/Abstract])) OR (Ventilations, Mechanical[Title/Abstract])) OR (Mechanical Ventilation[Title/Abstract])) OR (Ventilated[Title/Abstract])) OR (mechanically ventilated[Title/Abstract])) OR (ventilation[Title/Abstract]))
11. ("Liquid Ventilation"[Mesh]) OR ((((((((((((Ventilation, Liquid[Title/Abstract]) OR (Liquid Assisted Ventilation[Title/Abstract])) OR (Assisted Ventilation, Liquid[Title/Abstract])) OR (Ventilation, Liquid Assisted[Title/Abstract])) OR (Total Liquid Ventilation[Title/Abstract])) OR (Liquid Ventilation, Total[Title/Abstract])) OR (Ventilation, Total Liquid[Title/Abstract])) OR (Fluorocarbon Ventilation[Title/Abstract])) OR (Ventilation, Fluorocarbon[Title/Abstract])) OR (Partial Liquid Ventilation[Title/Abstract])) OR (Liquid Ventilation, Partial[Title/Abstract])) OR (Ventilation, Partial Liquid[Title/Abstract]))
12. ("Ventilators, Mechanical"[Mesh]) OR (((((((((((Mechanical Ventilator[Title/Abstract]) OR (Mechanical Ventilators[Title/Abstract])) OR (Ventilator, Mechanical[Title/Abstract])) OR (Pulmonary Ventilators[Title/Abstract])) OR (Ventilators, Pulmonary[Title/Abstract])) OR (Pulmonary Ventilator[Title/Abstract])) OR (Respirators[Title/Abstract])) OR (Respirator[Title/Abstract])) OR (Ventilator, Pulmonary[Title/Abstract])) OR (Ventilators[Title/Abstract])) OR (Ventilator[Title/Abstract]))
13. ("Continuous Positive Airway Pressure"[Mesh]) OR ((((((((((((((((CPAP Ventilation[Title/Abstract]) OR (Ventilation, CPAP[Title/Abstract])) OR (Nasal Continuous Positive Airway Pressure[Title/Abstract])) OR (nCPAP Ventilation[Title/Abstract])) OR (Ventilation, nCPAP[Title/Abstract])) OR (Airway Pressure Release Ventilation[Title/Abstract])) OR (APRV Ventilation Mode[Title/Abstract])) OR (APRV Ventilation Modes[Title/Abstract])) OR (Ventilation Mode, APRV[Title/Abstract])) OR (Ventilation Modes, APRV[Title/Abstract])) OR (Biphasic Continuous Positive Airway Pressure[Title/Abstract])) OR (BiPAP Biphasic Positive Airway Pressure[Title/Abstract])) OR (BiPAP Bilevel Positive Airway Pressure[Title/Abstract])) OR (Biphasic Positive Airway Pressure[Title/Abstract])) OR (Bilevel Continuous Positive Airway Pressure[Title/Abstract])) OR (Bilevel Positive Airway Pressure[Title/Abstract]))
14. ("Interactive Ventilatory Support"[Mesh]) OR ((((((Support, Interactive Ventilatory[Title/Abstract]) OR (Ventilatory Support, Interactive[Title/Abstract])) OR (Neurally Adjusted Ventilatory Assist[Title/Abstract])) OR (Proportional Assist Ventilation[Title/Abstract])) OR (Assist Ventilation, Proportional[Title/Abstract])) OR (Ventilation, Proportional Assist[Title/Abstract]))
15. **(Intermittent Positive-Pressure Breathing[MeSH Terms]) OR (((((((((Breathing, Intermittent Positive-Pressure[Title/Abstract]) OR (Intermittent Positive Pressure Breathing[Title/Abstract])) OR (Intermittent Positive Pressure Breathing[Title/Abstract])) OR (Intermittent Positive Pressure Breathing (IPPB[Title/Abstract]))) OR (Inspiratory Positive-Pressure Breathing[Title/Abstract])) OR (Breathing, Inspiratory Positive-Pressure[Title/Abstract])) OR (Inspiratory Positive Pressure Breathing[Title/Abstract])) OR (Positive-Pressure Breathing, Inspiratory[Title/Abstract])) OR (IPPB[Title/Abstract]))**
16. ("High-Frequency Ventilation"[Mesh]) OR ((((((((((((((((High-Frequency Ventilations[Title/Abstract]) OR (Ventilations, High-Frequency[Title/Abstract])) OR (Ventilation, High Frequency[Title/Abstract])) OR (Ventilation, High-Frequency[Title/Abstract])) OR (High Frequency Ventilation[Title/Abstract])) OR (High Frequency Ventilations[Title/Abstract])) OR (Ventilations, High Frequency[Title/Abstract])) OR (High-Frequency Oscillation Ventilation[Title/Abstract])) OR (High-Frequency Oscillation Ventilations[Title/Abstract])) OR (Oscillation Ventilation, High-Frequency[Title/Abstract])) OR (Oscillation Ventilations, High-Frequency[Title/Abstract])) OR (Ventilation, High-Frequency Oscillation[Title/Abstract])) OR (Ventilations, High-Frequency Oscillation[Title/Abstract])) OR (High Frequency Oscillation Ventilation[Title/Abstract])) OR (High-Frequency Positive Pressure Ventilation[Title/Abstract])) OR (High Frequency Positive Pressure Ventilation[Title/Abstract]))
17. ("Positive-Pressure Respiration"[Mesh]) OR ((((((((((((((((Positive Pressure Respiration[Title/Abstract]) OR (Positive Pressure Respirations[Title/Abstract])) OR (Respiration, Positive-Pressure[Title/Abstract])) OR (Respirations, Positive-Pressure[Title/Abstract])) OR (Positive-Pressure Ventilation[Title/Abstract])) OR (Positive Pressure Ventilation[Title/Abstract])) OR (Positive-Pressure Ventilations[Title/Abstract])) OR (Ventilation, Positive-Pressure[Title/Abstract])) OR (Ventilations, Positive-Pressure[Title/Abstract])) OR (Positive End-Expiratory Pressure[Title/Abstract])) OR (End-Expiratory Pressure, Positive[Title/Abstract])) OR (End-Expiratory Pressures, Positive[Title/Abstract])) OR (Positive End Expiratory Pressure[Title/Abstract])) OR (Positive End-Expiratory Pressures[Title/Abstract])) OR (Pressure, Positive End-Expiratory[Title/Abstract])) OR (Pressures, Positive End-Expiratory[Title/Abstract]))
18. ("Airway Extubation"[Mesh]) OR (((((((((((((((airway extubation[Title/Abstract]) OR (Extubation, Airway[Title/Abstract])) OR (Extubations, Airway[Title/Abstract])) OR (Tracheal Extubation[Title/Abstract])) OR (Extubation, Tracheal[Title/Abstract])) OR (Extubations, Tracheal[Title/Abstract])) OR (tracheal extubation[Title/Abstract])) OR (Extubation, Intratracheal[Title/Abstract])) OR (Extubations, Intratracheal[Title/Abstract])) OR (Intratracheal Extubation[Title/Abstract])) OR (Intratracheal Extubations[Title/Abstract])) OR (Endotracheal Extubation[Title/Abstract])) OR (Endotracheal Extubations[Title/Abstract])) OR (Extubation, Endotracheal[Title/Abstract])) OR (Extubations, Endotracheal[Title/Abstract]))
19. ("Intubation, Intratracheal"[Mesh]) OR (((((((Intratracheal Intubation[Title/Abstract]) OR (Intratracheal Intubations[Title/Abstract])) OR (Intubations, Intratracheal[Title/Abstract])) OR (Intubation, Endotracheal[Title/Abstract])) OR (Endotracheal Intubation[Title/Abstract])) OR (Endotracheal Intubations[Title/Abstract])) OR (Intubations, Endotracheal[Title/Abstract]))
20. **or/10-19**
21. **9 and 20**
22. ("Analgesics, Opioid"[Mesh]) OR (((((((((((((((((((((Opioid Analgesics[Title/Abstract]) OR (Opioid Analgesic[Title/Abstract])) OR (Analgesic, Opioid[Title/Abstract])) OR (Opioids[Title/Abstract])) OR (Opioid[Title/Abstract])) OR (Partial Opioid Agonists[Title/Abstract])) OR (Agonists, Partial Opioid[Title/Abstract])) OR (Opioid Agonists, Partial[Title/Abstract])) OR (Opioid Partial Agonists[Title/Abstract])) OR (Agonists, Opioid Partial[Title/Abstract])) OR (Partial Agonists, Opioid[Title/Abstract])) OR (Full Opioid Agonists[Title/Abstract])) OR (Agonists, Full Opioid[Title/Abstract])) OR (Opioid Agonists, Full[Title/Abstract])) OR (Opioid Full Agonists[Title/Abstract])) OR (Agonists, Opioid Full[Title/Abstract])) OR (Full Agonists, Opioid[Title/Abstract])) OR (Opioid Mixed Agonist-Antagonists[Title/Abstract])) OR (Agonist-Antagonists, Opioid Mixed[Title/Abstract])) OR (Mixed Agonist-Antagonists, Opioid[Title/Abstract])) OR (Opioid Mixed Agonist Antagonists[Title/Abstract]))
23. ("Analgesics"[Mesh]) OR ((((((((Anodynes[Title/Abstract]) OR (Analgesic Drugs[Title/Abstract])) OR (Drugs, Analgesic[Title/Abstract])) OR (Analgesic[Title/Abstract])) OR (Analgesic Agents[Title/Abstract])) OR (Agents, Analgesic[Title/Abstract])) OR (Antinociceptive Agents[Title/Abstract])) OR (Agents, Antinociceptive[Title/Abstract]))
24. ("Remifentanil"[Mesh]) OR (((((((3-(4-Methoxycarbonyl-4-((1-oxopropyl)phenylamino)-1-piperidine)propanoic Acid Methyl Ester[Title/Abstract]) OR (Remifentanil Hydrochloride[Title/Abstract])) OR (Ultiva[Title/Abstract])) OR (Remifentanil Monohydrochloride[Title/Abstract])) OR (GI 87084B[Title/Abstract])) OR (GI87084B[Title/Abstract])) OR (GI-87084B[Title/Abstract]))
25. ("Fentanyl"[Mesh]) OR ((((((((((((((((((((((((((((((((Phentanyl[Title/Abstract]) OR (Fentanest[Title/Abstract])) OR (Fentanyl Citrate[Title/Abstract])) OR (R-4263[Title/Abstract])) OR (R 4263[Title/Abstract])) OR (R4263[Title/Abstract])) OR (Sublimaze[Title/Abstract])) OR (Transmucosal Oral Fentanyl Citrate[Title/Abstract])) OR (Duragesic[Title/Abstract])) OR (Durogesic[Title/Abstract])) OR (Fentora[Title/Abstract])) OR (Abstral[Title/Abstract])) OR (Durotep[Title/Abstract])) OR (Fentamyl[Title/Abstract])) OR (Fentanylum[Title/Abstract])) OR (Fentora[Title/Abstract])) OR (IONSYS[Title/Abstract])) OR (Lazanda[Title/Abstract])) OR (Leptanal[Title/Abstract])) OR (Matrifen[Title/Abstract])) OR (Mhentanyl[Title/Abstract])) OR (Onsolis[Title/Abstract])) OR (Pecfent[Title/Abstract])) OR (Phentanyl[Title/Abstract])) OR (Rapinyl[Title/Abstract])) OR (Recuvyra[Title/Abstract])) OR (R-4263[Title/Abstract])) OR (Sublimase[Title/Abstract])) OR (Sublimaze[Title/Abstract])) OR (Subsys[Title/Abstract])) OR (Tanyl[Title/Abstract])) OR (transfenta[Title/Abstract]))
26. ("Sufentanil"[Mesh]) OR ((((((((((((((((Sulfentanyl[Title/Abstract]) OR (Sulfentanil[Title/Abstract])) OR (Sufenta[Title/Abstract])) OR (Sufentanil-Ratiopharm[Title/Abstract])) OR (Sufentanil Ratiopharm[Title/Abstract])) OR (SufentanilRatiopharm[Title/Abstract])) OR (Sufentanil Curasan[Title/Abstract])) OR (Curasan, Sufentanil[Title/Abstract])) OR (Sufentanil-Hameln[Title/Abstract])) OR (Sufentanil Hameln[Title/Abstract])) OR (SufentanilHameln[Title/Abstract])) OR (R-30730[Title/Abstract])) OR (R 30730[Title/Abstract])) OR (R30730[Title/Abstract])) OR (Sufentanil Citrate[Title/Abstract])) OR (Citrate, Sufentanil[Title/Abstract])
27. ("Morphine"[Mesh]) OR ((((((((((((((((((((((((((((((((((((Morphia[Title/Abstract]) OR (Morphine Chloride[Title/Abstract])) OR (Chloride, Morphine[Title/Abstract])) OR (Morphine Sulfate[Title/Abstract])) OR (Sulfate, Morphine[Title/Abstract])) OR (SDZ 202-250[Title/Abstract])) OR (SDZ 202 250[Title/Abstract])) OR (SDZ 202250[Title/Abstract])) OR (SDZ202-250[Title/Abstract])) OR (SDZ202 250[Title/Abstract])) OR (SDZ202250[Title/Abstract])) OR (Morphine Sulfate (2:1), Pentahydrate[Title/Abstract])) OR (MS Contin[Title/Abstract])) OR (Contin, MS[Title/Abstract])) OR (Oramorph SR[Title/Abstract])) OR (Duramorph[Title/Abstract])) OR (Depodur[Title/Abstract])) OR (Dimorf[Title/Abstract])) OR (Duromorph[Title/Abstract])) OR (Epimorph[Title/Abstract])) OR (l-Morphine[Title/Abstract])) OR (M-Eslon[Title/Abstract])) OR (Morphine Sulfate (2:1), Anhydrous[Title/Abstract])) OR (Aguettant[Title/Abstract])) OR (Miro[Title/Abstract])) OR (Morfina[Title/Abstract])) OR (Morphin[Title/Abstract])) OR (Morphina[Title/Abstract])) OR (Morphinum[Title/Abstract])) OR (Morphium[Title/Abstract])) OR (Moscontin[Title/Abstract])) OR (Nepenthe[Title/Abstract])) OR (Opso[Title/Abstract])) OR (Roxanol[Title/Abstract])) OR (Sevredol[Title/Abstract])) OR (skenan[Title/Abstract]))
28. **or/22-27**
29. **21 and 28**
30. **(clinical[tiab] AND trial[tiab]) OR "clinical trials as topic"[mesh] OR "clinical trial"[pt] OR random*[tiab] OR "random allocation"[mesh] OR "therapeutic use"[sh]**
31. **(animals[mh]) NOT (humans[mh])**
32. **(((Editorial[pt]) OR (Letter[pt])) OR (Case Reports[pt])) OR (Comment[pt])**
33. **30 not 31 not 32**
34. **29 and 33**
