# Supplementary material for: ICU patients receiving remifentanil do not experience reduced duration of mechanical ventilation: a systematic review of randomized controlled trials and network meta-analyses based on Bayesian theories
Source: Front Med (Lausanne). 2024 Aug 7;11:1370481. doi: 10.3389/fmed.2024.1370481 (PMC11342801; doi:10.3389/fmed.2024.1370481)
Supplement: Supplementary file 2 [file Data_Sheet_2.DOC]

# Additional file 2

**Description of included studies**

## Table S 2.1 Description of mechanical ventilation mode in the included studies

| **ID** | **Author/year** | **Mechanical ventilation mode** |
| --- | --- | --- |
| 1 | Yamush/1997 | NR |
| 2 | Chinachoti/2002 | NR |
| 3 | Dahaba/2004 | NR |
| 4 | Karabinis/2004 | NR |
| 5 | Muellejans/2004 | NR |
| 6 | Akinci/2005 | NR |
| 7 | Baillard/2005 | The patient ventilates spontaneously with pressure support. |
| 8 | Amor/2007 | NR |
| 9 | Carrer/2007 | Patients underwent mechanical pressure ventilation (biphasic intermittent positive airway pressure, BIPAP) and at regular intervals, were reassured on their condition. |
| 10 | Spies/2010 | NR |
| 11 | Cevik/2011 | Simultaneously Intermediate Mechanical Ventilation/Volume Control (SIMV/VC) mode |
| 12 | Oliver/2011 | Intermittent mandatory ventilation adjusted to maintain aP_a_CO_2_ level of 32 to 42 mmHg |
| 13 | Liu/2013 | NR |
| 14 | Lee/2014 | Controlled mode |
| 15 | Yang/2014 | NR |
| 16 | Yue/2016 | NR |
| 17 | Liu/2017 | NR |
| 18 | Casamento/2021 | NR |
| 19 | Doi/2023 | NR |
| 20 | Li/2023 | NR |

**Abbreviation:** NR: not reported

## Table S 2.2 Criteria of weaning from mechanical ventilation in the included studies

| **ID** | **Author/year** | **Criteria weaning from mechanical ventilation** |
| --- | --- | --- |
| 1 | Yamush/1997 | NR |
| 2 | Chinachoti/2002 | NR |
| 3 | Dahaba/2004 | Heart rate was < 100 beats/min, arterial partial pressure of oxygen was > 60 mm Hg with fractional inspired oxygen concentration < 0.5, arterial partial pressure of carbon dioxide (PaCO_2_) was < 45 mm Hg with minute volume < 101, and patients required no further sedative medications. |
| 4 | Karabinis/2004 | Judged by the investigator to be eligible to begin the wear process. |
| 5 | Muellejans/2004 | NR |
| 6 | Akinci/2005 | NR |
| 7 | Baillard/2005 | A spontaneous T-bump test was performed twice a day |
| 8 | Amor/2007 | NR |
| 9 | Carrer/2007 | NR |
| 10 | Spies/2010 | NR |
| 11 | Cevik/2011 | NR |
| 12 | Oliver/2011 | (1) mediastinal chest tube drainage below 3 mL/kg/h; (2) hemodynamic stability; (3) urine output exceeding 1 mL/g/h; (4) normothermia (>36°C); (5) pH ≥7.35 and ≤7.55, arterial carbon dioxide tension (PaCO_2_) ≥30 mmHg and ≤50 mmHg, arterial oxygen tension (PaO_2_) ≥80 mmHg, and respiratory rate ≤25/min; and (6) absence of residual neuromuscular blockade according to train-of-4 response with stimulation of the ulnar nerve. |
| 13 | Liu/2013 | NR |
| 14 | Lee/2014 | a. Oxygenation: PaO_2_ > 60 mmHg with FiO_2_ < 0.4, PaO_2_/FiO_2_ > 150, SaO_2_ > 90%, PEEP < 5 cmH2O, and minute volume < 15 L/min.  b. Vital signs: mean arterial pressure > 60 mmHg without vasopressor, heart rate < 140/min, 35°C < body temperature < 38°C, and respiratory rate < 35/min.  c. Clinical status: resolution of acute disease phase, no newly developed definite pulmonary infiltration, Ramsay sedation score 2-4, hemoglobin > 7 g/dl, pH > 7.30, electrolyte within normal range, no active bleeding, no increased intracranial pressure, no bronchospasm, no untreated coronary arterial disease, and no specific treatment such as nitric oxide gas, prone position or operation plan etc. |
| 15 | Yang/2014 | NR |
| 16 | Yue/2016 | NR |
| 17 | Liu/2017 | NR |
| 18 | Casamento/2021 | NR |
| 19 | Doi/2023 | NR |
| 20 | Li/2023 | NR |

**Abbreviation:** NR: not reported

## Table S 2.3 Description of extubation criteria in the included studies

| **ID** | **Author/year** | **Extubation criteria** |
| --- | --- | --- |
| 1 | Yamush/1997 | NR |
| 2 | Chinachoti/2002 | NR |
| 3 | Dahaba/2004 | Patients were responsive to simple commands, their respiratory rate was 10–16 breaths/min with spontaneous tidal volume > 5 ml/kg. |
| 4 | Karabinis/2004 | Judged by the investigator to be eligible to begin the extubation process. |
| 5 | Muellejans/2004 | Qualification of patients for extubation was at the discretion of individual investigators, based upon their clinical judgement. |
| 6 | Akinci/2005 | Judged by the investigator to be eligible to begin the extubation process. |
| 7 | Baillard/2005 | The patient is able to perform spontaneous T-shaped ventilation for two hours, with a breathing rate of less than 35 breaths/min, a maximum inspiratory pressure of less than -20 cmH_2_O, and a tidal volume of more than 5 ml/kg. If the tube is not reintubated within 48 hours after extubation, the weaning is considered successful. |
| 8 | Amor/2007 | NR |
| 9 | Carrer/2007 | By the ICU physician in conformity with the usual criteria. |
| 10 | Spies/2010 | NR |
| 11 | Cevik/2011 | NR |
| 12 | Oliver/2011 | When criteria to begin weaning the ventilator were satisfied and a Ramsay sedation level ≤3 was present, an intensivist and respiratory therapist, unaware of a patient’s treatment group, began weaning the ventilator toward eventual tracheal extubation according to a standardized protocol |
| 13 | Liu/2013 | NR |
| 14 | Lee/2014 | NR |
| 15 | Yang/2014 | NR |
| 16 | Yue/2016 | NR |
| 17 | Liu/2017 | NR |
| 18 | Casamento/2021 | NR |
| 19 | Doi/2023 | NR |
| 20 | Li/2023 | NR |

**Abbreviation:** NR: not reported
