# Supplementary material for: ICU patients receiving remifentanil do not experience reduced duration of mechanical ventilation: a systematic review of randomized controlled trials and network meta-analyses based on Bayesian theories
Source: Front Med (Lausanne). 2024 Aug 7;11:1370481. doi: 10.3389/fmed.2024.1370481 (PMC11342801; doi:10.3389/fmed.2024.1370481)

# Additional file 3

**Results from network meta-analyses**

The size of the nodes corresponds to the total number of participants that study the treatments. The (directly) comparable treatments are linked with a line. The thickness of the line corresponds to the standard error of trials that study this comparison. The colours of the line corresponds to the quality of trials that study this comparison. low risk of bias [green] ,moderate risk of bias [yellow].

## Figure S 3.1 Network plot of all intervention comparisons for duration of extubation


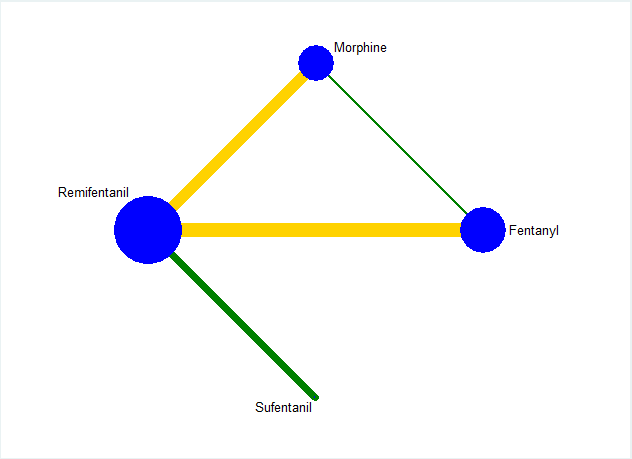


## Figure S 3.2 Network plot of all intervention comparisons for ICU length of stay


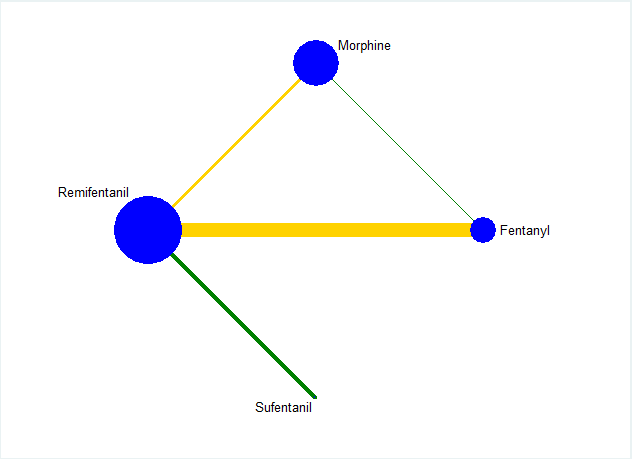


## Figure S 3.3 Network plot of all intervention comparisons for ICU mortality


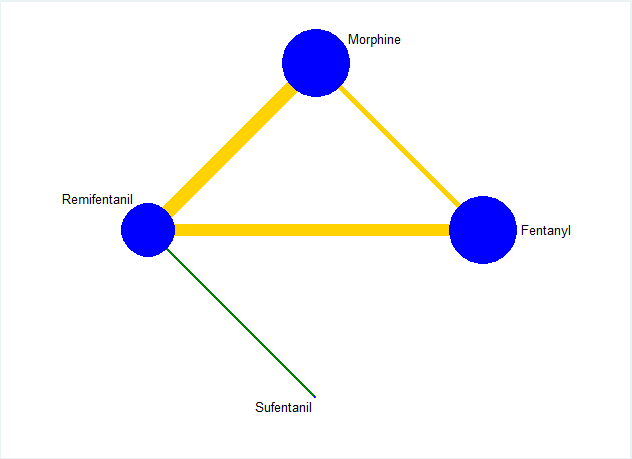


## Figure S 3.4 Network plot of all intervention comparisons for efficacy


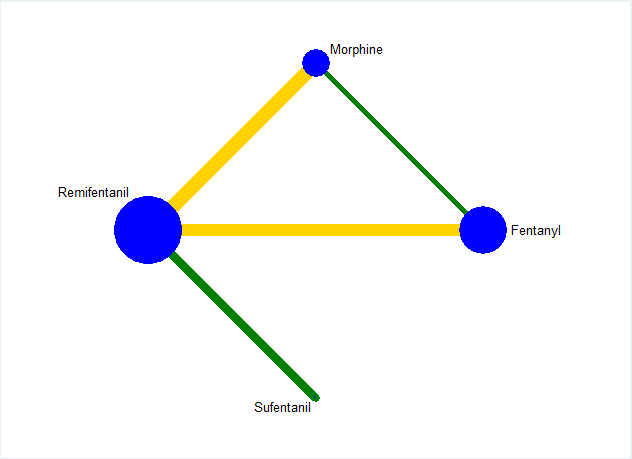


## Figure S 3.5 Network plot of all intervention comparisons for safety


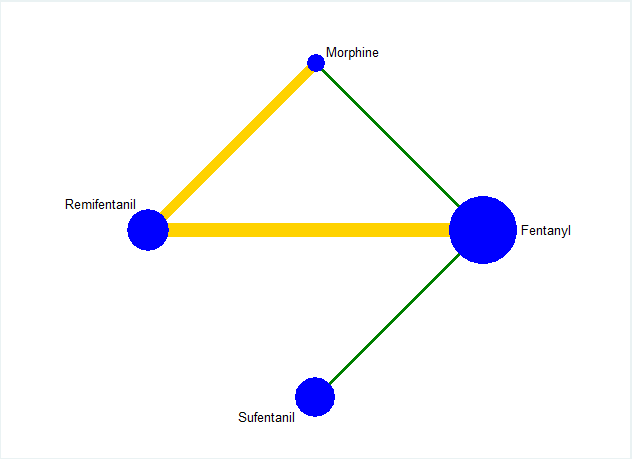


## Figure S 3.6 Network plot of all intervention comparisons for drug-related hypotensive


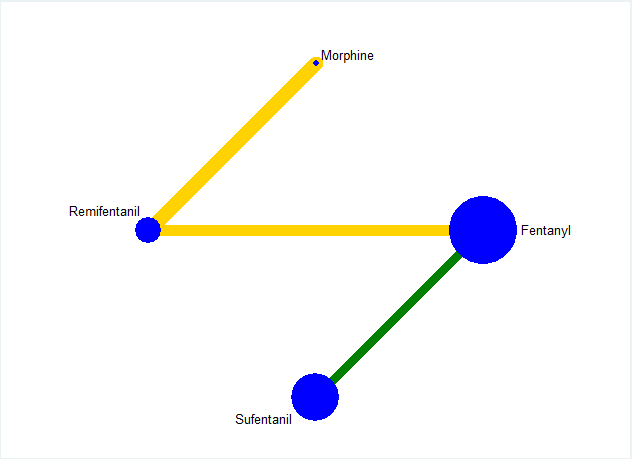


## Figure S 3.7 Network plot of all intervention comparisons for drug-related bradycardia


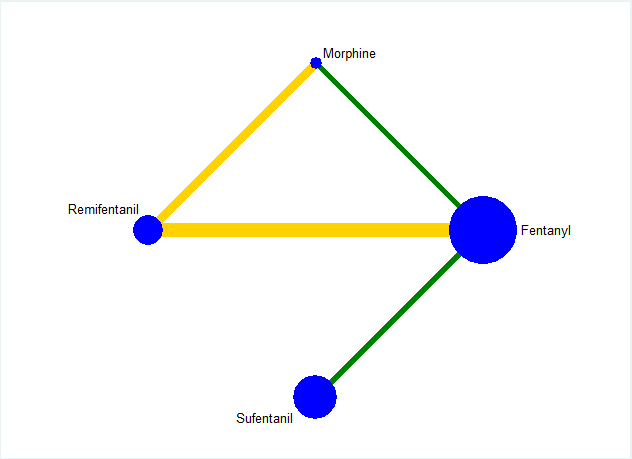


## Figure S 3.8 Network plot of all intervention comparisons for drug-related bradypnea


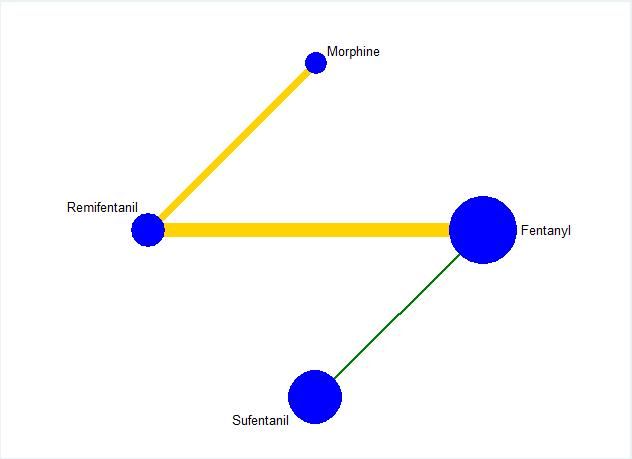

Supplement: Supplementary file 3 [file Data_Sheet_3.DOC]
