# Supplementary material for: ICU patients receiving remifentanil do not experience reduced duration of mechanical ventilation: a systematic review of randomized controlled trials and network meta-analyses based on Bayesian theories
Source: Front Med (Lausanne). 2024 Aug 7;11:1370481. doi: 10.3389/fmed.2024.1370481 (PMC11342801; doi:10.3389/fmed.2024.1370481)
Supplement: Supplementary file 4 [file Data_Sheet_4.DOC]

# Additional file 4

**Assessment of heterogeneity in treatment for each outcome network**

We assess heterogeneity between direct comparisons and global heterogeneity by looked at the *I*^2^ weather it is high compared to the expected value (50%). The following figure all show the results of heterogeneity between direct comparisons and golbal in each outcome network. The study number in the third column of table corresponds to the serial number of the references in table 1. F: Fentanyl; M: Morphine; R: Remifentanil; S: Sufentanil

# Table S 4.1 Assessment of heterogeneity in networks

| **Outcome** | **Treatment** | **Study** | **Per-comparison**  **I-squared(%)** | | **Global**  **I-squared(%)** | |
| --- | --- | --- | --- | --- | --- | --- |
|  |  |  | **Pair-wise** | **Network** | **Pair** | **Compairson** |
| Duration of MV | M vs F | 4, 18 | -- | 81.2 | 59.95 | 67.70 |
|  | R vs F | 4,5,8,10,11,13,17,19,20 | 19.5 | 14.5 |  |  |
|  | R vs M | 2,3,4,9 | 78.5 | 85.1 |  |  |
|  | | | | | | |
| Duration of extubation | M vs F | 4,12 | 0 | 0 | 32.65 | 35.78 |
|  | R vs F | 4,5,6,8,17,19,20 | 49.5 | 43.1 |  |  |
|  | R vs M | 1,2,3,4,14 | 21.8 | 7.74 |  |  |
|  | S vs R | 7 | -- | -- |  |  |
|  | | | | | | |
| ICU length of stay | M vs F | 4 |  | 31.5 | 99.85 | 99.87 |
|  | R vs F | 4,5,8,10,11,13,17,20 | 0.1 | 0 |  |  |
|  | R vs M | 1,3,4,9,12,18 | 100 | 100 |  |  |
|  | S vs R | 7 | -- | -- |  |  |
|  | | | | | | |
| ICU mortality | M vs F | 4,18 | -- | -- | 0 | 0 |
|  | R vs F | 4,19,20 | -- | -- |  |  |
|  | R vs M | 2,3,4,9 | 0 | 0 |  |  |
|  | S vs R | 7 | -- | -- |  |  |
|  | | | | | | |
| Efficiay | M vs F | 4 | -- | 49.6 | 62.04 | 71.91 |
|  | R vs F | 4,5,19,20 | 0 | 28 |  |  |
|  | R vs M | 2,3,4 | 72.6 | 82.6 |  |  |
|  | S vs R | 7 | -- | -- |  |  |
|  | | | | | | |
| Safety | M vs F | 4 | -- | 0 | 0 | 0 |
|  | R vs F | 4,5,11,13,19,20 | 7 | 7.1 |  |  |
|  | S vs F | 15,16 | 1.6 | 1.1 |  |  |
|  | R vs M | 2,3,4,9 | 0 | 0 |  |  |
|  | | | | | | |
| Hpotensive | R vs F | 5,6,11,13,20 | 20.8 | 20.7 | 2.57 | 2.41 |
|  | S vs F | 15,16 | 0 | 0 |  |  |
|  | R vs M | 3,9 | 10.5 | 9.9 |  |  |
|  | | | | | | |
| Badycardia | M vs F | 4 | -- | 0 | 73.39 | 21.78 |
|  | R vs F | 4,5,11,13,20 | 80.6 | 50.6 |  |  |
|  | S vs F | 15,16 | 0 | 0 |  |  |
|  | R vs M | 4,9 | 67.4 | 22.8 |  |  |
|  | | | | | | |
| Badypnea | M vs F | 4,18 | 0 | 0 | 0 | 0 |
|  | R vs F | 4,5,8,10,11,13,17,19 | 0 | 0 |  |  |
|  | R vs M | 2,3,4,9 | 0 | 0 |  |  |

**Assessment of inconsistency in treatment for each outcome network**

We use Node-splitting analysis to assess inconsistencies between direct and indirect comparisons, and‘design-by-treatment’ interaction model to assess global inconsistencies (p<0.05). F: Fentanyl; M: Morphine; R: Remifentanil;

## Table S 4.2 Assesment of inconsistency in networks

| **Outcome** | **Node-splitting analysis of inconsistency** | |  | **Global inconsistency** | |
| --- | --- | --- | --- | --- | --- |
|  | **Comparison** | **P value** |  | **Chi-square** | **P value** |
| Duration of MV | M vs F | 0.0979 |  | 5.51 | 0.0635 |
|  |  |  |  |  |  |
| Duration of extubation | M vs F | 0.720 |  | 1.53 | 0.6745 |
|  | R vs F | 0.610 |  |  |  |
|  | R vs M | 0.711 |  |  |  |
|  |  |  |  |  |  |
| ICU length of stay | M vs F | 0.401 |  | 1.02 | 0.6005 |
|  |  |  |  |  |  |
| ICU mortality | M vs F | 0.050 |  | 0.10 | 0.7560 |
|  |  |  |  |  |  |
| Efficiay | M vs F | 0.497 |  | 0.97 | 0.6170 |
|  |  |  |  |  |  |
| Safety | M vs F | 0.979 |  | 1.87 | 0.3923 |
|  |  |  |  |  |  |
| Badycardia | M vs F | 0.4081 |  | 0.02 | 0.9895 |
