# Supplementary material for: ICU patients receiving remifentanil do not experience reduced duration of mechanical ventilation: a systematic review of randomized controlled trials and network meta-analyses based on Bayesian theories
Source: Front Med (Lausanne). 2024 Aug 7;11:1370481. doi: 10.3389/fmed.2024.1370481 (PMC11342801; doi:10.3389/fmed.2024.1370481)
Supplement: Supplementary file 5 [file Data_Sheet_5.DOC]

# Additional file 5

**Assessment of transitivity**

# Figure S 5.1 Transitivity in the network of primary outcome


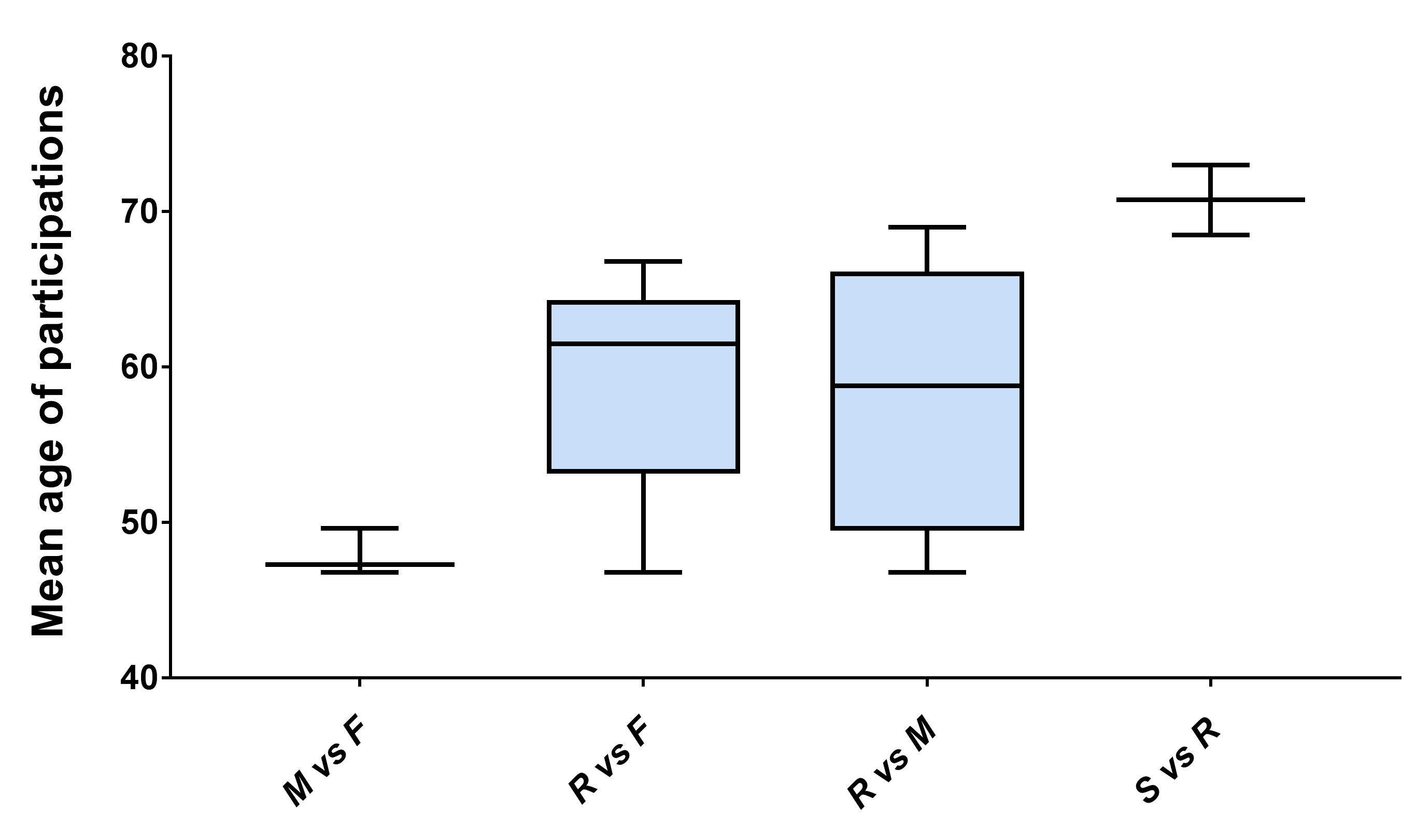


F: Fentanyl; M: Morphine; R: Remifentanil; S: Sufentanil
