# Supplementary material for: ICU patients receiving remifentanil do not experience reduced duration of mechanical ventilation: a systematic review of randomized controlled trials and network meta-analyses based on Bayesian theories
Source: Front Med (Lausanne). 2024 Aug 7;11:1370481. doi: 10.3389/fmed.2024.1370481 (PMC11342801; doi:10.3389/fmed.2024.1370481)
Supplement: Supplementary file 6 [file Data_Sheet_6.DOC]

# Additional file 6

**Comparison-adjusted funnel plot for each outcome form the network meta-analysis**

# Figure S 6.1 Comparison-adjusted funnel plot for the network of duration of mechanical ventilation in all comparisons


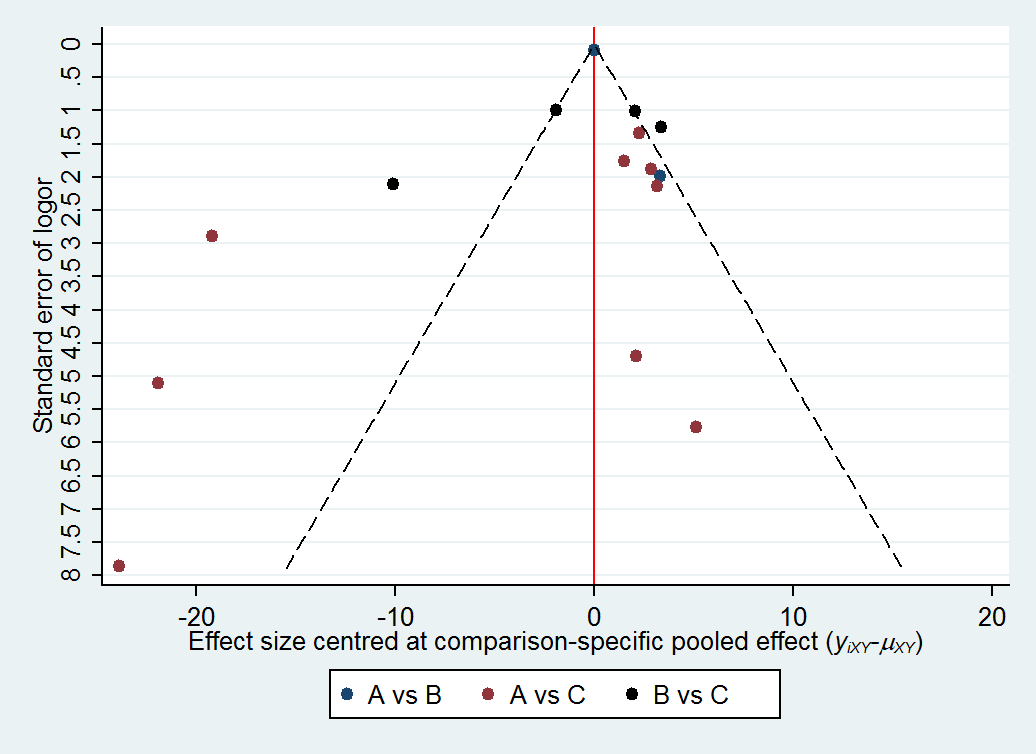


A: Fentanyl; B: Morphine; C: Remifentanil

# Figure S6.2 Comparison-adjusted funnel plot for the network of duration of extubation in all comparisons


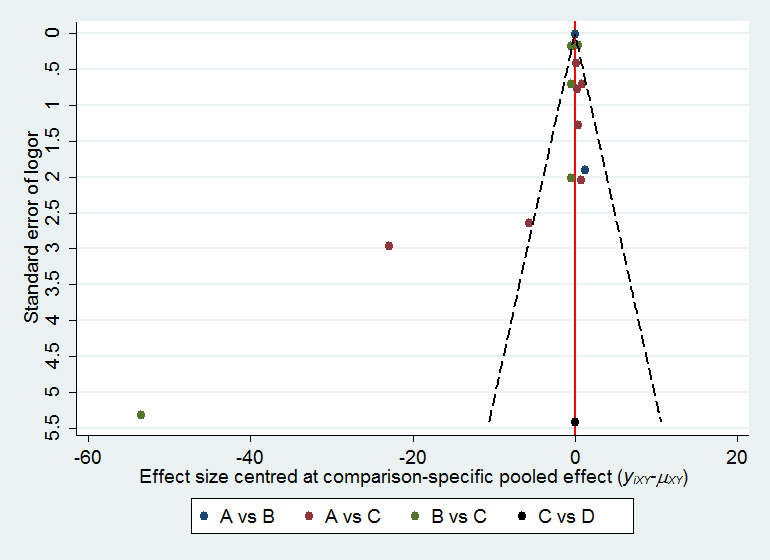


A: Fentanyl; B: Morphine; C: Remifentanil; D: Sufentanil

# Figure S 6.3 Comparison-adjusted funnel plot for the network of ICU length of stay in all comparisons


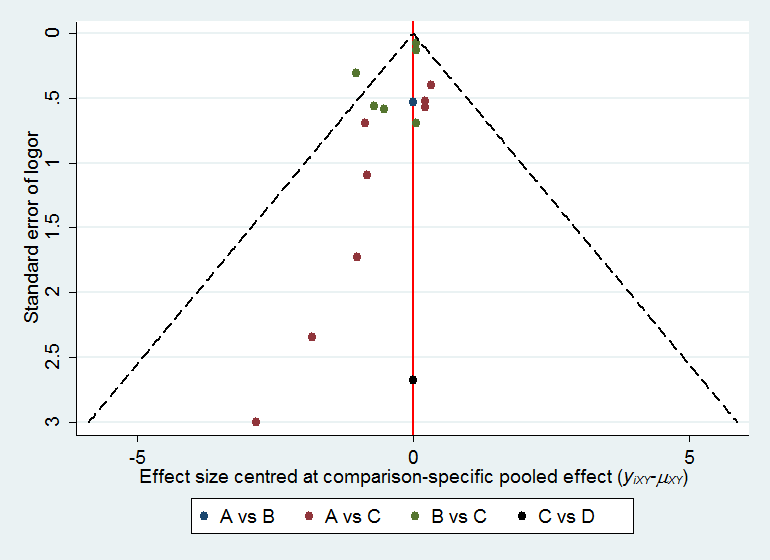


A: Fentanyl; B: Morphine; C: Remifentanil; D: Sufentanil

# Figure S 6.4 Comparison-adjusted funnel plot for the network of ICU mortality in all comparisons


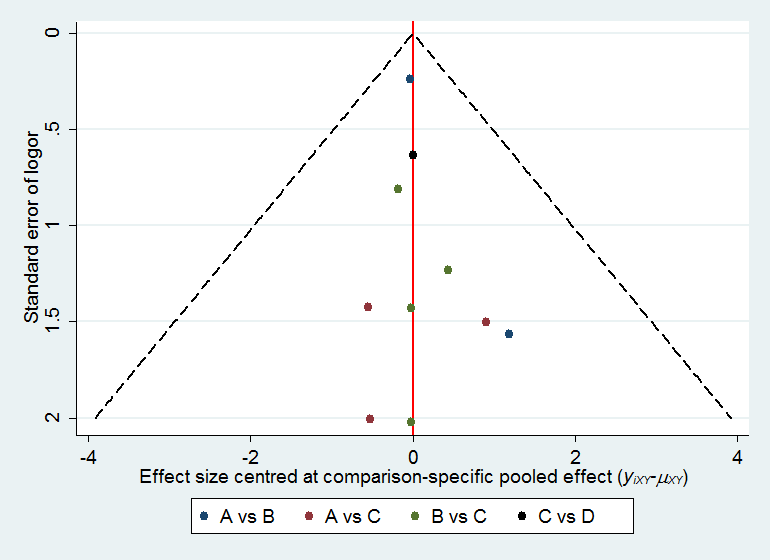


A: Fentanyl; B: Morphine; C: Remifentanil; D: Sufentanil

# Figure S 6.5 Comparison-adjusted funnel plot for the network of efficacy in all comparisons


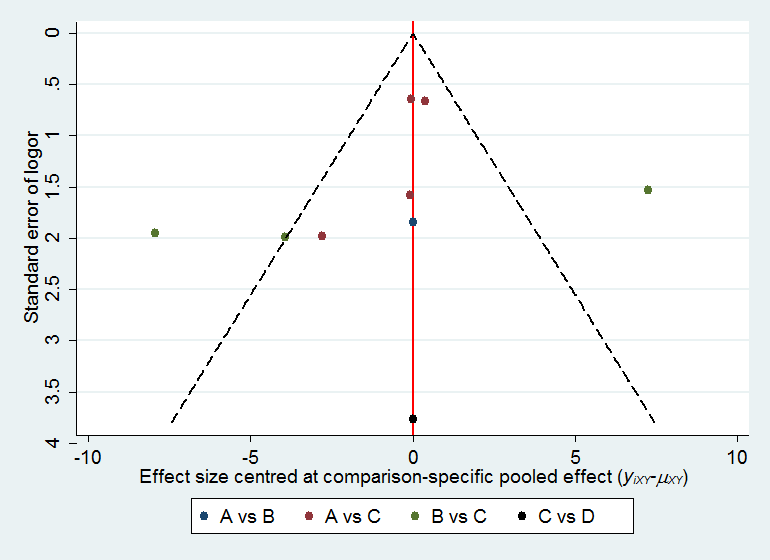


A: Fentanyl; B: Morphine; C: Remifentanil; D: Sufentanil

# Figure S 6.6 Comparison-adjusted funnel plot for the network of safety in all comparisons


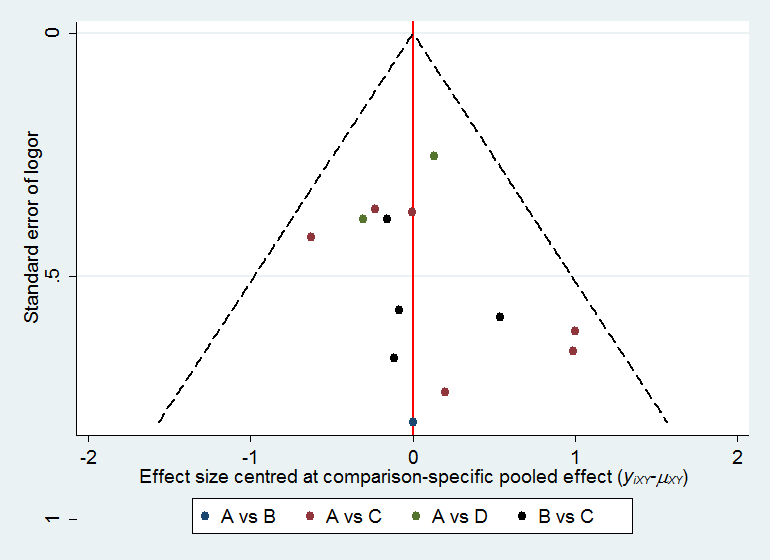


A: Fentanyl; B: Morphine; C: Remifentanil; D: Sufentanil

# Figure S 6.7 Comparison-adjusted funnel plot for the network of hypotensive related to remifentanil in all comparisons


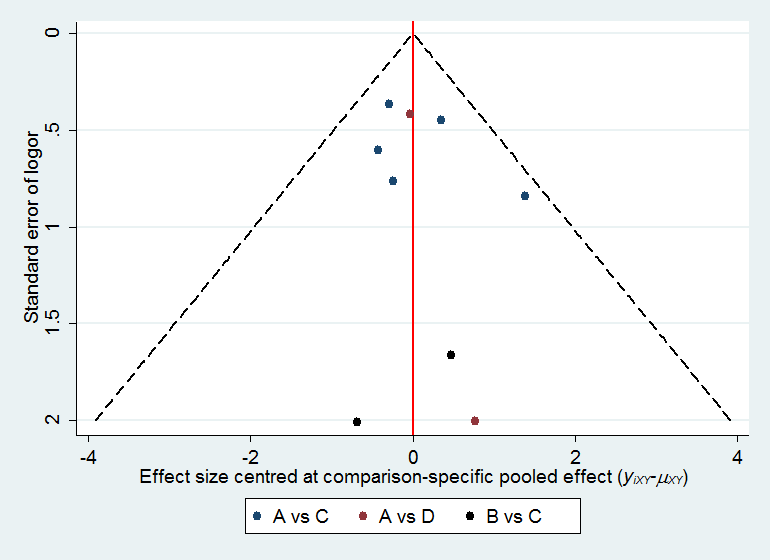


A: Fentanyl; B: Morphine; C: Remifentanil; D: Sufentanil

# Figure S 6.8 Comparison-adjusted funnel plot for the network of bradycardia related to remifentanil in all comparisons


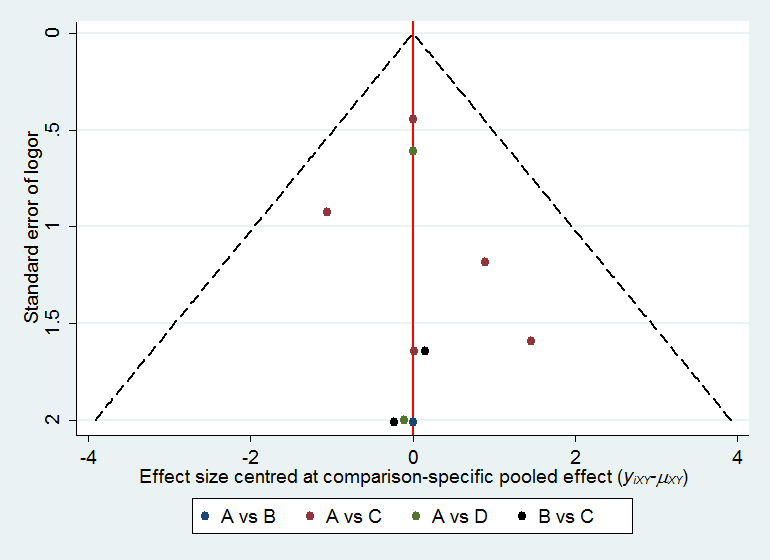


A: Fentanyl; B: Morphine; C: Remifentanil; D: Sufentanil

# Figure S 6.9 Comparison-adjusted funnel plot for the network of bradypnea related to remifentanil in all comparisons


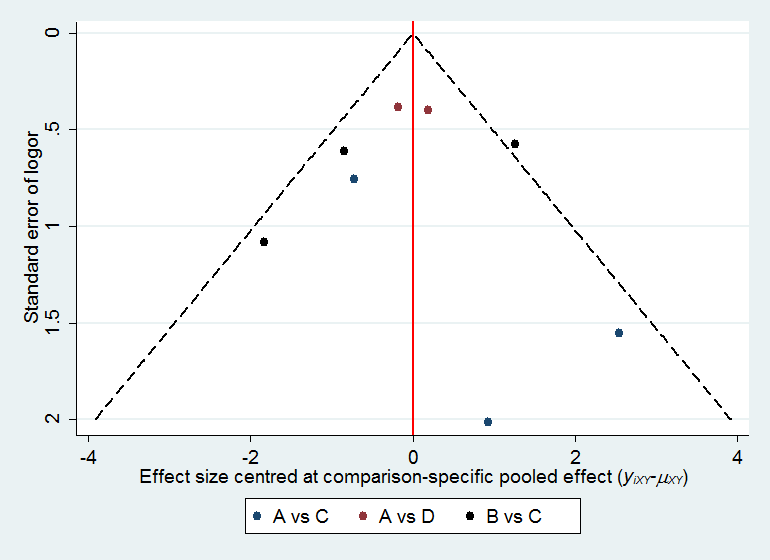


A: Fentanyl; B: Morphine; C: Remifentanil; D: Sufentanil
