# Supplementary material for: ICU patients receiving remifentanil do not experience reduced duration of mechanical ventilation: a systematic review of randomized controlled trials and network meta-analyses based on Bayesian theories
Source: Front Med (Lausanne). 2024 Aug 7;11:1370481. doi: 10.3389/fmed.2024.1370481 (PMC11342801; doi:10.3389/fmed.2024.1370481)
Supplement: Supplementary file 7 [file Data_Sheet_7.DOC]

# Additional file 7

**GRADE for the primary and second outcomes**

Based on the above assessment of RoB for each comparison and the contribution matrix detailing contribution of each direct comparison to all network estimates, the following bar graphs show the percentage of low or moderate or high RoB contributions for each network estimate.

The judgements about study limitations in each direct comparison is shown at the beginning of the graph. Each bar corresponds to a NMA relative treatment effect and shows how much information comes from comparisons at low risk of bias [green] or moderate risk of bias [yellow].

Based on all the above information, we GRADEd each network estimate according to the following criteria.

(1)**Study limitations**: We downgraded by one level when the contributions from low RoB comparisons were less than 30% and contributions from moderate RoB comparisons were 70% or greater.

(2)**Imprecision**: We considered a clinically meaningful threshold for OR to be 0.80 or 1.25 and downgraded the estimate if the OR point estimate is 1 or more and the lower limit of its CrI is below 0.80; or if the OR point estimate is less than 1 and the upper limit of its CrI is above 1.25.

(3)**Inconsistency**: We rated two concepts, heterogeneity and incoherence (inconsistency), in this domain. For heterogeneity, we looked at the *I*^2^ and found weather it is high compared to the expected value (50%). For inconsistency, we looked at the results of side splitting and ‘design-by-treatment’ interaction model. We did downgraded the comparisons with important inconsistency (p<0.05) (we could downgrade the same network estimate for both heterogeneity and inconsistency).

(4)**Indirectness**: We have assured transitivity in our network by limiting the included studies to critically ill adult patients. We assured that they did not violate transitivity of the network. Hence, we did not downgraded singly-connected nodes for indirectness because evaluation of transitivity for such nodes is clear.

(5)**Publication bias**: We managed to retrieve supplementary and unpublished information included in the available systematic reviews and network meta-analyses, and we assessed publication bias using the comparison-adjusted funnel plots

# Table S 7.1 Result of GRADE for duration of MV

|  | **Nature of the evidence** | **Study**  **limitations** | **Imprecision** | **Inconsistency** | **Indirectness** | **Publication**  **bias** | **Confidence** | Downgrading due to |
| --- | --- | --- | --- | --- | --- | --- | --- | --- |
| A vs B | Mixed estimated | No downgrade | Downgrade because point estimate < 1.0 but upper limit >1.25 | Downgrade because pair heterogeneity *I*^2^=81.2% | No downgrade | No downgrade | LOW | Imprecision Inconsistency - |
| A vs C | Mixed estimated | No downgrade | Downgrade because point estimate >1.0 but lower limit<0.80 | No downgrade | No downgrade | Downgrade because publication bias | LOW | Imprecision Publication bias |
| B vs C | Mixed estimated | Downgrade because ＞70% contribution from moderate Rob comparisons | Downgrade because point estimate >1.0 but lower limit<0.80 | Downgrade because pair heterogeneity *I*^2^=85.1% | No downgrade | Downgrade because publication bias | VERY LOW | Study limitations Imprecision  Inconsistency  Publication bias |
| Ranking of treatments |  | No downgrade | Downgrade because similar distributions of ranks | Downgrade because global heterogeneity *I*^2^=67.70% | No downgrade | Downgrade because publication bias | VERY LOW | Imprecision  Inconsistency  Publication bias |

A: Fentanyl; B: Morphine; C: Remifentanil;

# Table S 7.2 Result of GRADE for duration of extubation

|  | **Nature of the evidence** | **Study**  **limitations** | **Imprecision** | **Inconsistency** | **Indirectness** | **Publication**  **bias** | **Confidence** | Downgrading due to |
| --- | --- | --- | --- | --- | --- | --- | --- | --- |
| A vs B | Mixed estimated | Downgrade because ＞70% contribution from moderate Rob comparisons | No downgrade | No downgrade | No downgrade | No downgrade | MODERATE | Study limitations |
| A vs C | Mixed estimated | Downgrade because ＞70% contribution from moderate Rob comparisons | Downgrade because point estimate < 1.0 but upper limit >1.25 | No downgrade | No downgrade | Downgrade because publication bias | VERY LOW | Study limitations Imprecision  Publication bias |
| A vs D | Indirect estimated | Downgrade because ＞70% contribution from moderate Rob comparisons | No downgrade | No downgrade | No downgrade | No downgrade | MODERATE | Study limitations |
| B vs C | Mixed estimated | Downgrade because ＞70% contribution from moderate Rob comparisons | Downgrade because point estimate < 1.0 but upper limit >1.25 | No downgrade | No downgrade | Downgrade because publication bias | VERY LOW | Study limitations Imprecision  Publication bias |
| B vs D | Indirect estimated | Downgrade because ＞70% contribution from moderate Rob | No downgrade | No downgrade | No downgrade | No downgrade | MODERATE | Study limitations |
| C vs D | Mixed estimated | Downgrade because ＞70% contribution from moderate Rob comparisons | No downgrade | No downgrade | No downgrade | No downgrade | MODERATE | Study limitations |
| Ranking of treatments |  | Downgrade because ＞70% contribution from moderate Rob comparisons | No downgrade | No downgrade | No downgrade | Downgrade because publication bias | LOW | Study limitations  Publication bias |

A: Fentanyl; B: Morphine; C: Remifentanil; D: Sufentanil;

# Table S 7.3 Result of GRADE for ICU length of stay

|  | **Nature of the evidence** | **Study**  **limitations** | **Imprecision** | **Inconsistency** | **Indirectness** | **Publication**  **bias** | **Confidence** | Downgrading due to |
| --- | --- | --- | --- | --- | --- | --- | --- | --- |
| A vs B | Mixed estimated | Downgrade because ＞70% contribution from moderate Rob comparisons | No downgrade | No downgrade | No downgrade | No downgrade | MODERATE | Study limitations |
| A vs C | Mixed estimated | Downgrade because ＞70% contribution from moderate Rob comparisons | No downgrade | No downgrade | No downgrade | No downgrade | MODERATE | Study limitations |
| A vs D | Indirect estimated | Downgrade because ＞70% contribution from moderate Rob comparisons | Downgrade because point estimate >1.0 but lower limit<0.80 | No downgrade | No downgrade | No downgrade | LOW | Study limitations Imprecision |
| B vs C | Mixed estimated | Downgrade because ＞70% contribution from moderate Rob comparisons | Downgrade because point estimate < 1.0 but upper limit >1.25 | Downgrade because pair heterogeneity *I*^2^=100% | No downgrade | No downgrade | VERY LOW | Study limitations Imprecision Inconsistency |
| B vs D | Indirect estimated | Downgrade because ＞70% contribution from moderate Rob comparisons | Downgrade because point estimate >1.0 but lower limit<0.80 | No downgrade | No downgrade | No downgrade | LOW | Study limitations Imprecision |
| C vs D | Mixed estimated | Downgrade because ＞70% contribution from moderate Rob comparisons | Downgrade because point estimate >1.0 but lower limit<0.80 | No downgrade | No downgrade | No downgrade | LOW | Study limitations Imprecision |
| Ranking of treatments |  | Downgrade because ＞70% contribution from moderate Rob comparisons | Downgrade because similar distributions of ranks | Downgrade because global heterogeneity *I*^2^=99.9% | No downgrade | Downgrade | VERY LOW | Study limitations Imprecision Inconsistency |

A: Fentanyl; B: Morphine; C: Remifentanil; D: Sufentanil;

# Table S 7.4 Result of GRADE for ICU mortality

|  | **Nature of the evidence** | **Study**  **limitations** | **Imprecision** | **Inconsistency** | **Indirectness** | **Publication**  **bias** | **Confidence** | Downgrading due to |
| --- | --- | --- | --- | --- | --- | --- | --- | --- |
| A vs B | Indirect estimated | No downgrade | No downgrade | Downgrade because node-splitting analysis of inconsistency *p*=0.05 | No downgrade | No downgrade | MODERATE | Inconsistency - |
| A vs C | Mixed estimated | No downgrade | No downgrade | No downgrade | No downgrade | No downgrade | HIGH | -- |
| A vs D | Indirect estimated | No downgrade | No downgrade | No downgrade | No downgrade | No downgrade | HIGH | -- |
| B vs C | Mixed estimated | Downgrade because ＞70% contribution from moderate Rob comparisons | Downgrade because point estimate < 1.0 but upper limit >1.25 | No downgrade | No downgrade | No downgrade | LOW | Study limitations Imprecision |
| B vs D | Indirect estimated | Downgrade because ＞70% contribution from moderate Rob | Downgrade because point estimate < 1.0 but upper limit >1.25 | No downgrade | No downgrade | No downgrade | LOW | Study limitations Imprecision |
| C vs D | Mixed estimated | Downgrade because ＞70% contribution from moderate Rob comparisons | Downgrade because point estimate < 1.0 but upper limit >1.25 | No downgrade | No downgrade | No downgrade | LOW | Study limitations Imprecision |
| Ranking of treatments |  | No downgrade | Downgrade because similar distributions of ranks | No downgrade | No downgrade | No downgrade | MODERATE | Imprecision |

# Table S 7.5 Result of GRADE for efficacy

|  | **Nature of the evidence** | **Study**  **limitations** | **Imprecision** | **Inconsistency** | **Indirectness** | **Publication**  **bias** | **Confidence** | Downgrading due to |
| --- | --- | --- | --- | --- | --- | --- | --- | --- |
| A vs B | Mixed estimated | No downgrade | Downgrade because point estimate >1.0 but lower limit<0.80 | No downgrade | No downgrade | No downgrade | MODERATE | Imprecision- |
| A vs C | Mixed estimated | Downgrade because ＞70% contribution from moderate Rob comparisons | Downgrade because point estimate < 1.0 but upper limit >1.25 | No downgrade | No downgrade | No downgrade | LOW | Study limitations  Imprecision |
| A vs D | Indirect estimated | Downgrade because ＞70% contribution from moderate Rob comparisons | Downgrade because point estimate < 1.0 but upper limit >1.25 | No downgrade | No downgrade | No downgrade | LOW | Study limitations Imprecision |
| B vs C | Mixed estimated | No downgrade | Downgrade because point estimate < 1.0 but upper limit >1.25 | Downgrade because pair heterogeneity *I*^2^=82.6% | No downgrade | No downgrade | LOW | Imprecision  Inconsistency |
| B vs D | Indirect estimated | Downgrade because ＞70% contribution from moderate Rob | Downgrade because point estimate < 1.0 but upper limit >1.25 | No downgrade | No downgrade | No downgrade | LOW | Study limitations Imprecision |
| C vs D | Mixed estimated | Downgrade because ＞70% contribution from moderate Rob comparisons | Downgrade because point estimate < 1.0 but upper limit >1.25 | No downgrade | No downgrade | No downgrade | LOW | Study limitations  Imprecision |
| Ranking of treatments |  | Downgrade because ＞70% contribution from moderate Rob comparisons | Downgrade because similar distributions of ranks | Downgrade because global heterogeneity *I*^2^=71.91% | No downgrade | No downgrade | VERY LOW | Study limitations Imprecision  Inconsistency |

A: Fentanyl; B: Morphine; C: Remifentanil; D: Sufentanil;

# Table S 7.6 Result of GRADE for safety

|  | **Nature of the evidence** | **Study**  **limitations** | **Imprecision** | **Inconsistency** | **Indirectness** | **Publication**  **bias** | **Confidence** | Downgrading due to |
| --- | --- | --- | --- | --- | --- | --- | --- | --- |
| A vs B | Mixed estimated | Downgrade because ＞70% contribution from moderate Rob comparisons | Downgrade because point estimate < 1.0 but upper limit >1.25 | No downgrade | No downgrade | No downgrade | LOW | Study limitations Imprecision- |
| A vs C | Mixed estimated | Downgrade because ＞70% contribution from moderate Rob comparisons | No downgrade | No downgrade | No downgrade | No downgrade | MODERATE | Study limitations |
| A vs D | Mixed estimated | Downgrade because ＞70% contribution from moderate Rob comparisons | Downgrade because point estimate >1.0 but lower limit<0.80 | No downgrade | No downgrade | No downgrade | LOW | Study limitations Imprecision |
| B vs C | Mixed estimated | Downgrade because ＞70% contribution from moderate Rob comparisons | No downgrade | No downgrade | No downgrade | No downgrade | MODERATE | Study limitations |
| B vs D | Indirect estimated | Downgrade because ＞70% contribution from moderate Rob | Downgrade because point estimate >1.0 but lower limit<0.80 | No downgrade | No downgrade | No downgrade | LOW | Study limitations Imprecision |
| C vs D | Indirect estimated | Downgrade because ＞70% contribution from moderate Rob comparisons | No downgrade | No downgrade | No downgrade | No downgrade | MODERATE | Study limitations |
| Ranking of treatments |  | Downgrade because ＞70% contribution from moderate Rob comparisons | No downgrade | No downgrade | No downgrade | No downgrade | MODERATE | Study limitations |

A: Fentanyl; B: Morphine; C: Remifentanil; D: Sufentanil;

# Table S 7.7 Result of GRADE for hypotensive

|  | **Nature of the evidence** | **Study**  **limitations** | **Imprecision** | **Inconsistency** | **Indirectness** | **Publication**  **bias** | **Confidence** | Downgrading due to |
| --- | --- | --- | --- | --- | --- | --- | --- | --- |
| A vs B | Indirect estimated | Downgrade because ＞70% contribution from moderate Rob comparisons | Downgrade because point estimate >1.0 but lower limit<0.80 | No downgrade | No downgrade | No downgrade | LOW | Study limitations Imprecision |
| A vs C | Mixed estimated | Downgrade because ＞70% contribution from moderate Rob comparisons | Downgrade because point estimate < 1.0 but upper limit >1.25 | No downgrade | No downgrade | No downgrade | LOW | Study limitations Imprecision |
| A vs D | Mixed estimated | Downgrade because ＞70% contribution from moderate Rob comparisons | Downgrade because point estimate >1.0 but lower limit<0.80 | No downgrade | No downgrade | No downgrade | LOW | Study limitations Imprecision |
| B vs C | Mixed estimated | Downgrade because ＞70% contribution from moderate Rob comparisons | Downgrade because point estimate < 1.0 but upper limit >1.25 | No downgrade | No downgrade | No downgrade | LOW | Study limitations Imprecision |
| B vs D | Indirect estimated | Downgrade because ＞70% contribution from moderate Rob comparisons | Downgrade because point estimate < 1.0 but upper limit >1.25 | No downgrade | No downgrade | No downgrade | LOW | Study limitations Imprecision |
| C vs D | Indirect estimated | Downgrade because ＞70% contribution from moderate Rob comparisons | Downgrade because point estimate >1.0 but lower limit<0.80 | No downgrade | No downgrade | No downgrade | LOW | Study limitations Imprecision |
| Ranking of treatments |  | Downgrade because ＞70% contribution from moderate Rob comparisons | No downgrade | No downgrade | No downgrade | No downgrade | HIGH | - |

A: Fentanyl; B: Morphine; C: Remifentanil; D: Sufentanil;

# Table S 7.8 Result of GRADE for bradycardia

|  | **Nature of the evidence** | **Study**  **limitations** | **Imprecision** | **Inconsistency** | **Indirectness** | **Publication**  **bias** | **Confidence** | Downgrading due to |
| --- | --- | --- | --- | --- | --- | --- | --- | --- |
| A vs B | Mixed estimated | No downgrade | Downgrade because point estimate >1.0 but lower limit<0.80 | No downgrade | No downgrade | No downgrade | MODERATE | Study limitations |
| A vs C | Mixed estimated | No downgrade | Downgrade because point estimate < 1.0 but upper limit >1.25 | Downgrade because pair heterogeneity *I*^2^=50.6% | No downgrade | No downgrade | LOW | Imprecision Inconsistency |
| A vs D | Indirect estimated | Downgrade because ＞70% contribution from moderate Rob comparisons | Downgrade because point estimate < 1.0 but upper limit >1.25 | No downgrade | No downgrade | No downgrade | LOW | Study limitations Imprecision |
| B vs C | Mixed estimated | Downgrade because ＞70% contribution from moderate Rob | Downgrade because point estimate < 1.0 but upper limit >1.25 | No downgrade | No downgrade | No downgrade | LOW | Study limitations Imprecision |
| B vs D | Indirect estimated | No downgrade | Downgrade because point estimate < 1.0 but upper limit >1.25 | No downgrade | No downgrade | No downgrade | MODERATE | Imprecision |
| C vs D | Mixed estimated | No downgrade | Downgrade because point estimate >1.0 but lower limit<0.80 | No downgrade | No downgrade | No downgrade | MODERATE | Imprecision |
| Ranking of treatments |  | No downgrade | Downgrade because similar distributions of ranks | No downgrade | No downgrade | No downgrade | MODERATE | Imprecision |

A: Fentanyl; B: Morphine; C: Remifentanil; D: Sufentanil;

# Table S 7.9 Result of GRADE for bradypnea

|  | **Nature of the evidence** | **Study**  **limitations** | **Imprecision** | **Inconsistency** | **Indirectness** | **Publication**  **bias** | **Confidence** | Downgrading due to |
| --- | --- | --- | --- | --- | --- | --- | --- | --- |
| A vs B | Indirect estimated | Downgrade because ＞70% contribution from moderate Rob comparisons | Downgrade because point estimate >1.0 but lower limit<0.80 | No downgrade | No downgrade | No downgrade | LOW | Study limitations Imprecision |
| A vs C | Mixed estimated | Downgrade because ＞70% contribution from moderate Rob comparisons | Downgrade because point estimate >1.0 but lower limit<0.80 | No downgrade | No downgrade | No downgrade | LOW | Study limitations Imprecision |
| A vs D | Mixed estimated | Downgrade because ＞70% contribution from moderate Rob comparisons | Downgrade because point estimate >1.0 but lower limit<0.80 | No downgrade | No downgrade | No downgrade | LOW | Study limitations Imprecision |
| B vs C | Mixed estimated | Downgrade because ＞70% contribution from moderate Rob comparisons | No downgrade | No downgrade | No downgrade | No downgrade | MODERATE | Study limitations |
| B vs D | Indirect estimated | Downgrade because ＞70% contribution from moderate Rob comparisons | Downgrade because point estimate >1.0 but lower limit<0.80 | No downgrade | No downgrade | No downgrade | LOW | Study limitations Imprecision |
| C vs D | Indirect estimated | Downgrade because ＞70% contribution from moderate Rob comparisons | Downgrade because point estimate < 1.0 but upper limit >1.25 | No downgrade | No downgrade | No downgrade | LOW | Study limitations Imprecision |
| Ranking of treatments |  | Downgrade because ＞70% contribution from moderate Rob comparisons | Downgrade because similar distributions of ranks | No downgrade | No downgrade | No downgrade | LOW | Study limitations Imprecision |

A: Fentanyl; B: Morphine; C: Remifentanil; D: Sufentanil;
