# Supplementary material for: ICU patients receiving remifentanil do not experience reduced duration of mechanical ventilation: a systematic review of randomized controlled trials and network meta-analyses based on Bayesian theories
Source: Front Med (Lausanne). 2024 Aug 7;11:1370481. doi: 10.3389/fmed.2024.1370481 (PMC11342801; doi:10.3389/fmed.2024.1370481)
Supplement: Supplementary file 8 [file Data_Sheet_8.DOC]

# Additional file 8

**Treatment ranking and surface under the cumulative ranking curves (SUCRA) for each outcome**

## Figure S 8.1 Treatment ranking and SUCRA ranking curve for duration of mechanical ventalition


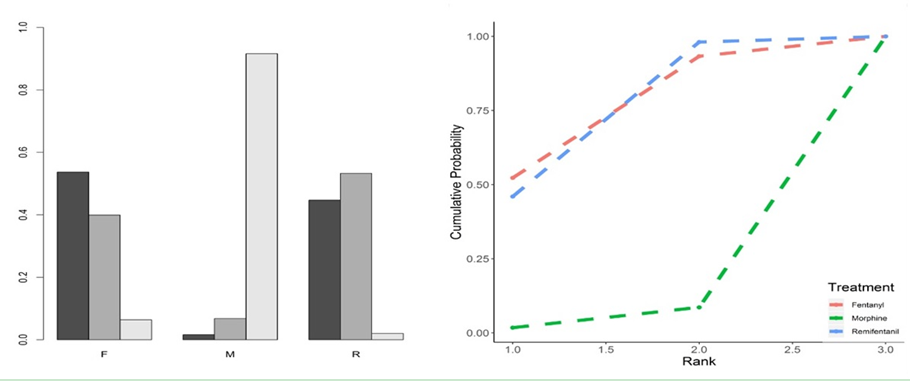


F: Fentanyl; M: Morphine; R: Remifentanil; S: Sufentanil

## Figure S 8.2 Treatment ranking and SUCRA ranking curve for duration of extubation


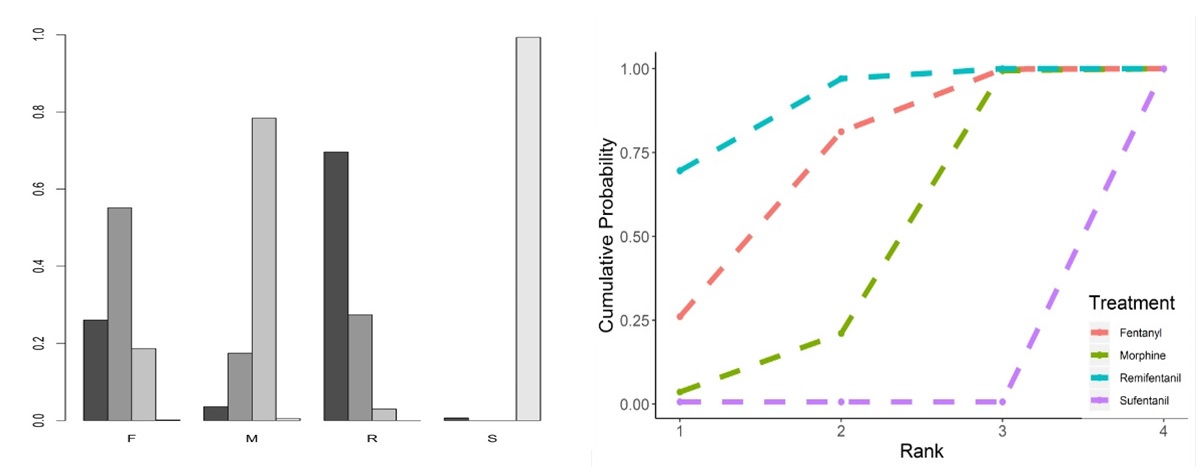


F: Fentanyl; M: Morphine; R: Remifentanil; S: Sufentanil

## Figure S 8.3 Treatment ranking and SUCRA ranking curve for ICU length of stay


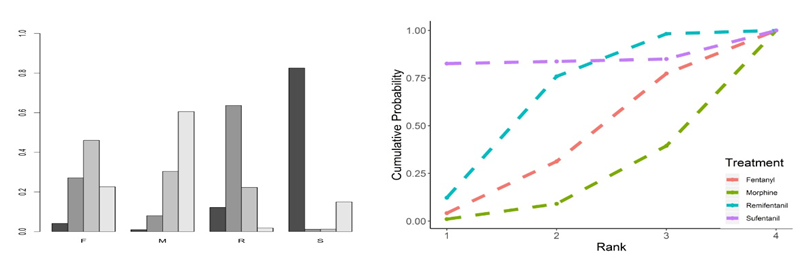


F: Fentanyl; M: Morphine; R: Remifentanil; S: Sufentanil

## Figure S 8.4 Treatment ranking and SUCRA ranking curve for ICU mortality


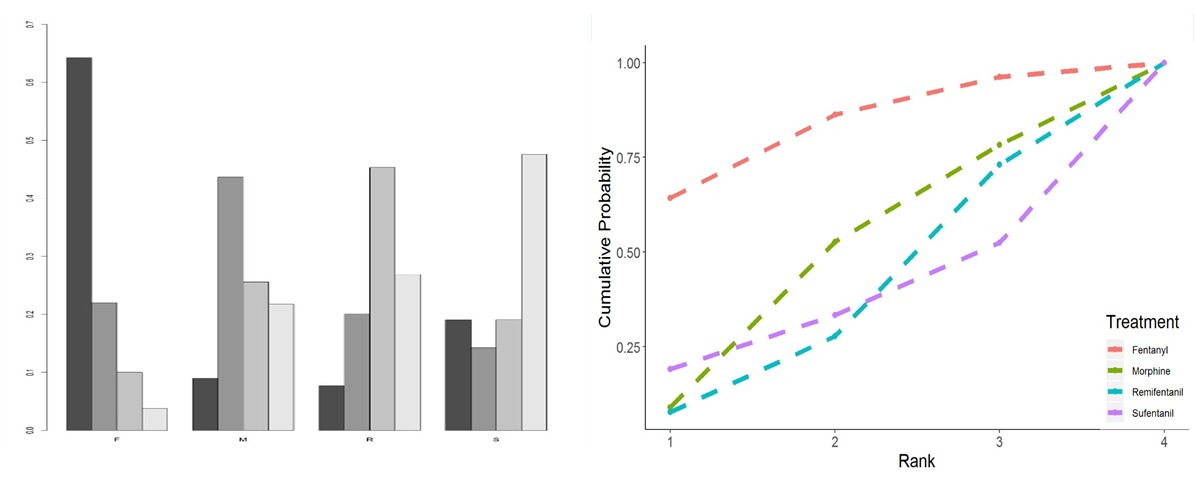


F: Fentanyl; M: Morphine; R: Remifentanil; S: Sufentanil

## Figure S 8.5 Treatment ranking and SUCRA ranking curve for efficacy


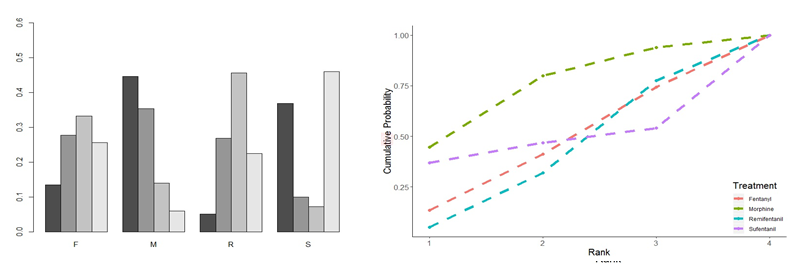


F: Fentanyl; M: Morphine; R: Remifentanil; S: Sufentanil

## Figure S 8.6 Treatment ranking and SUCRA ranking curve for safety


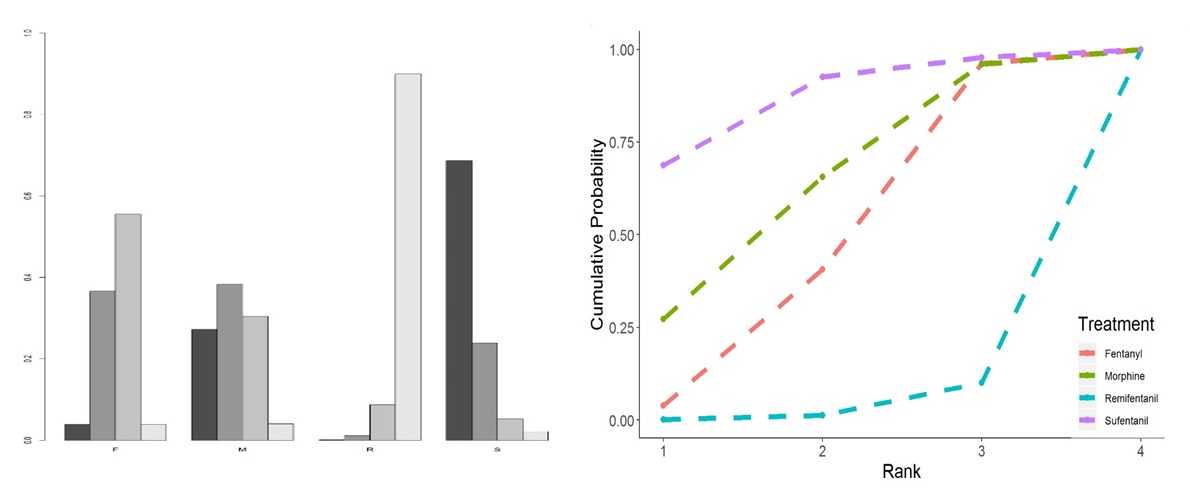


F: Fentanyl; M: Morphine; R: Remifentanil; S: Sufentanil

## Figure S 8.7 Treatment ranking and SUCRA ranking curve for hypotensive


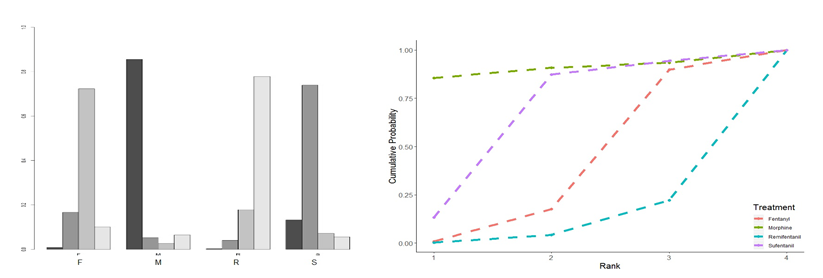


F: Fentanyl; M: Morphine; R: Remifentanil; S: Sufentanil

## Figure S 8.8 Treatment ranking and SUCRA ranking curve for bradycardia


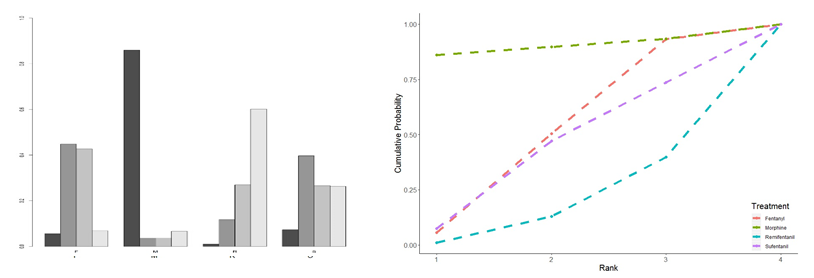


F: Fentanyl; M: Morphine; R: Remifentanil; S: Sufentanil

## Figure S 8.9 Treatment ranking and SUCRA ranking curve for bradypnea


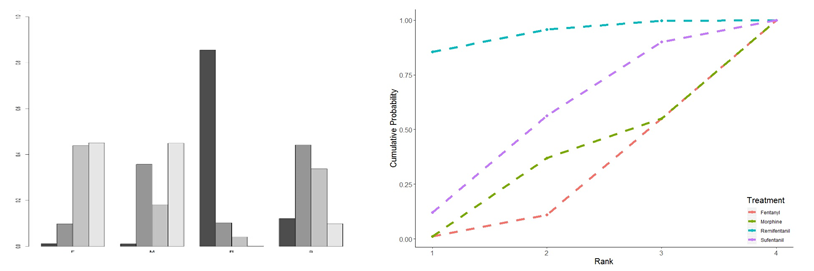


F: Fentanyl; M: Morphine; R: Remifentanil; S: Sufentanil
