# Supplementary material for: ICU patients receiving remifentanil do not experience reduced duration of mechanical ventilation: a systematic review of randomized controlled trials and network meta-analyses based on Bayesian theories
Source: Front Med (Lausanne). 2024 Aug 7;11:1370481. doi: 10.3389/fmed.2024.1370481 (PMC11342801; doi:10.3389/fmed.2024.1370481)
Supplement: Supplementary file 9 [file Data_Sheet_9.DOC]

# Additional file 9

**Sensitivity analyses**

## Table S 9.1 Sensitivity analyses for the risk of duration of mechanical ventilation

| **Treatment** | **Overall patients** | | **High quality studies only** | | **Studies without publication bias** | |
| --- | --- | --- | --- | --- | --- | --- |
|  | **MDs (95% CrI)** | **Rank** | **MDs (95% CrI)** | **Rank** | **MDs (95% CrI)** | **Rank** |
| **Fentanyl** | -0.16 (-4.75, 5.63) | 1 | -0.62 (-5.62, 4.09) | 1 | -0.83(-5.78, 3.71) | 1 |
| **Morphine** | 3.84 (-0.29, 10.68) | 3 | 2.48 (-1.47, 7.19) | 3 | 2.46(-1.42, 6.86) | 3 |
| **Remifentanil** | Reference | 2 | Reference | 2 | Reference | 2 |
| **Number of studies** | 13 | | 11 | | 9 | |
| **partcipants** | 1860 | | 1666 | | 1509 | |
